# Assessing microhabitat, landscape features and intraguild relationships in the occupancy of the enigmatic and threatened Andean tiger cat (*Leopardus tigrinus pardinoides*) in the cloud forests of northwestern Colombia

**DOI:** 10.1371/journal.pone.0288247

**Published:** 2023-07-10

**Authors:** Juan Camilo Cepeda-Duque, Gabriel Andrade-Ponce, Andrés Montes-Rojas, Uriel Rendón-Jaramillo, Valentina López-Velasco, Eduven Arango-Correa, Álex López-Barrera, Luis Mazariegos, D. Diego Lizcano, Andrés Link, Tadeu Gomes de Oliveira

**Affiliations:** 1 Laboratorio de Ecología de Bosques Tropicales, Departamento de Ciencias Biológicas, Universidad de los Andes, Bogotá, Colombia; 2 Red de Biología y Conservación de Vertebrados, Instituto de Ecología, A.C., Xalapa, Veracruz, México; 3 Fundación Bioconservancy, Jardín, Antioquia, Colombia; 4 Programa de Biología, Universidad del Quindío, Armenia, Quindío, Colombia; 5 Grupo de Investigación en Biología de la Conservación y Biotecnología, Corporación Universitaria de Santa Rosa de Cabal, Santa Rosa de Cabal, Risaralda, Colombia; 6 Fundación Caipora, Cajicá, Cundinamarca, Colombia; 7 Departamento de Biologia, Universidade Estadual do Maranhão (UEMA), Campus Paulo VI, São Luís, Maranhão, Brazil; 8 Programa de Pós-Graduação em Ecologia e Conservação da Biodiversidade PPGECB/PPG Em Ciência Animal da Universidade Estadual Do Maranhão, Cidade Universitária Paulo VI, São Luís, MA, Brazil; University of Glasgow, UNITED KINGDOM

## Abstract

Mesocarnivores play a key role in ecosystem dynamics through the regulation of prey populations and are sensitive to environmental changes; thus, they are often considered good model organisms for conservation planning. However, data regarding the factors that influence the habitat use of threatened small wild felids such as the Andean tiger cat (*Leopardus tigrinus pardinoides*) are scarce. We conducted a two-year survey with 58 camera trap stations to evaluate the determinants of Andean tiger cat habitat use in three protected areas in the Middle Cauca, Colombia. We developed site occupancy models and found that Andean tiger cat habitat use increased with leaf litter depth at intermediate elevations and far from human settlements. Through conditional cooccurrence models, we found that Andean tiger cat habitat use was invariant to the presence of prey or potential intraguild competitors and killers/predators, but its detectability increased when they were present and detected. This suggests that Andean tiger cats may be more likely to be detected in sites with high prey availability. We found that Andean tiger cats preferred sites with deep leaf litter, which is a particular feature of cloud forests that provides suitable conditions for ambush hunting and hiding from intraguild enemies. Our results showed that Andean tiger cats avoided human settlements, which may minimize potential mortality risks in those areas. Moreover, the restricted use of middle elevations by Andean tiger cats suggested that they could be used as a sentinel species to track the effects of climate change since their suitable habitat is likely to be projected upward in elevation. Future conservation actions must be focused on identifying and mitigating human-related threats close to the Andean tiger cat habitat while preserving microhabitat conditions and the existing networks of protected areas.

## Introduction

Small wild felids are considered keystone organisms for biodiversity maintenance, as they control the population growth of a wide range of small vertebrates that may include seed predators, invasive species, or disease vectors [1, 2]. The abundance and distribution of small wild felids are often mediated either by habitat quantity and quality or by interspecific interactions with prey and competitors, including humans [3]. Greater habitat structural complexity has been associated with increased hunting success and more effective avoidance of intraguild competitors/predators [4–6]. The spatiotemporal availability of prey represents a gradient of opportunities to supply metabolic requirements and successfully raise offspring [7]. Dominant carnivores may reduce the fitness of mesocarnivores through prey competition, intraguild predation/interspecific killing (nonnutritional benefit), or both [8–11]. However, partitioning resources (*e*.*g*., space, time, or food) is the most common way for small wild felids to coexist with superior competitors and killers/predators [8, 9]. Small wild felids are further constrained by human activities, such as deforestation and direct persecution for poultry depredation, and by the invasion of their habitats by domestic species, such as dogs [11, 12].

**Fig 1:**
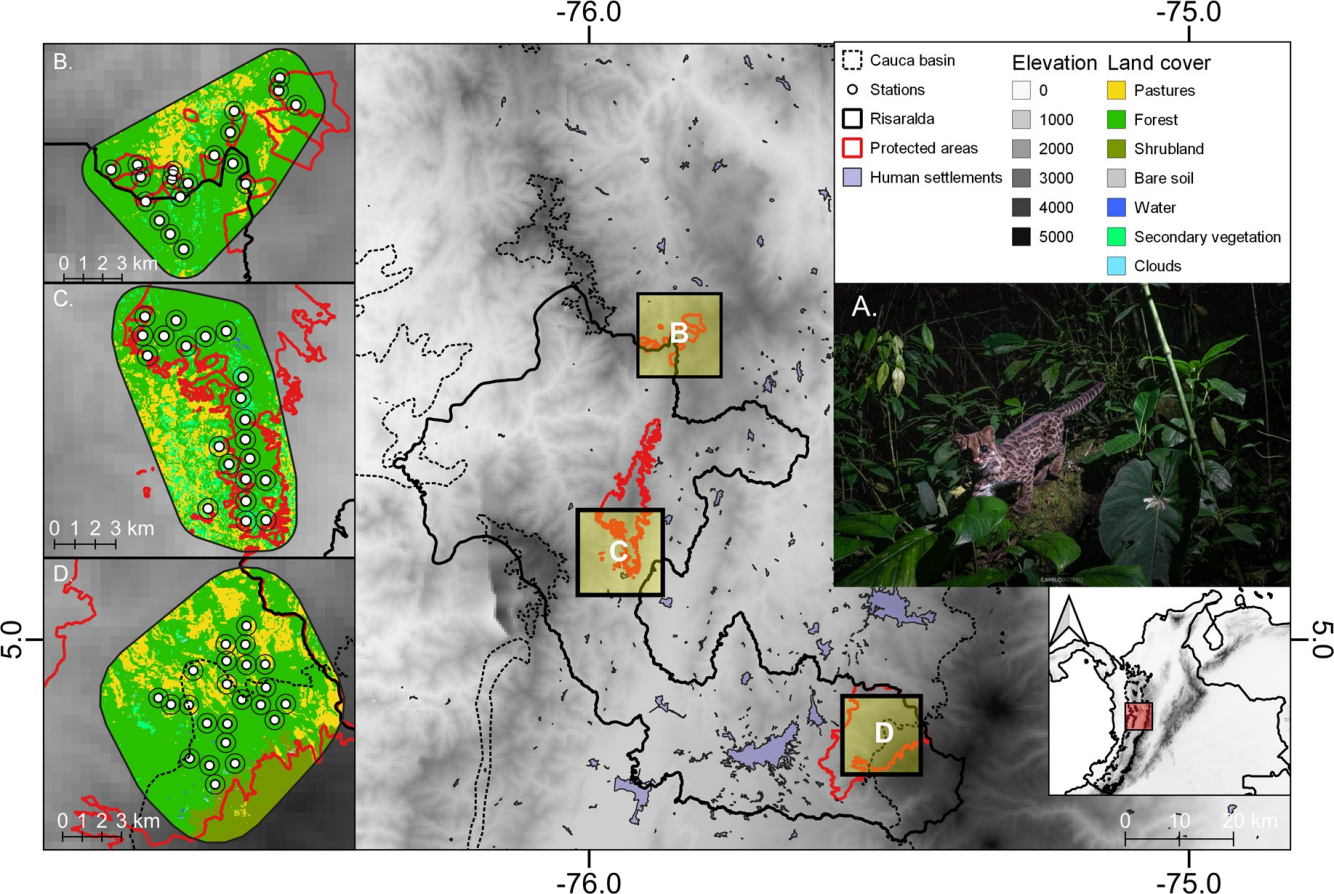
Andean tiger cat in its conservation stronghold, the Andean cloud forest of the Middle Cauca (A), and protected areas where this research was conducted. The Mesenia-Paramillo Nature Reserve – MPNR (B), the Cuchilla de San Juan Integrated Management District – CSJIMD (C) and the Campoalegre Soil Conservation District – CSCD (D). Represented are the main types of land covers and the Risaralda department, the target national land division unit. Layers of land cover types, elevation, and human settlements were obtained from their original sources, which are available online [38–40].

The Andean tiger cat (*Leopardus tigrinus pardinoides*) is a small mesocarnivore (weight: ca. 2.2 kg, length: 70-80 cm; Fig 1A) distributed in northwestern South America [13]. Tiger cats mainly hunt small mammals, reptiles, and birds [2]. Little is known about the basic natural history of these animals, especially the spatial and temporal interactions of Andean tiger cats with sympatric carnivores and their prey [14, 15]. They are found in small and large forest remnants [15, 16] and in pristine and disturbed areas [17]. The Andean tiger cat is categorized as vulnerable, and its populations are declining across its geographic range worldwide [17] and specifically in Colombia [18]. Andean tiger cat occurrence in Colombia is thought to be mainly constrained by both natural (inter-Andean valleys below 1500 m asl) and anthropogenic barriers (human settlements and roads) [19, 20]. The latter are specifically associated with the legacy of an intensive human footprint that has impacted the northwestern Andes region since the 1970s, a period of rampant population and economic growth in Colombia [21]. Conversely, it has been suggested that shielding the habitat of this forest-dwelling felid with integrative networks of public and private protected areas could promote the conditions for its persistence [16]. Nevertheless, important knowledge gaps regarding which drivers are relevant to infer the habitat use of the Andean tiger cat remain in the region and must be filled to strengthen conservation actions.

Camera traps are a well-stablished tool for studying relevant population parameters such as abundance, density, and occupancy of a diverse range of cryptic or elusive species [22]. In this context, occupancy has been used as a robust method for determining the role of habitat features, interspecific interactions, and human activities in the occurrence of poorly understood species [23]. In Colombia, there have been a growing number of studies assessing the effects of different abiotic and biotic predictors on the occupancy of small wild felids, such as ocelots (*Leopardus pardalis*), jaguarundis (*Herpailurus yagouaroundi*), margays (*L*. *wiedii*), and Andean tiger cats [15, 16, 24–26]. Only two occupancy studies involving the elusive Andean tiger cat in mountain cloud forests have been carried out using single and multispecies approaches. Notably, the conditional effect of food and risk availability, at least in some form of cooccurrence between Andean tiger cats and both small mammals and other sympatric carnivores, respectively, is still overlooked. However, it could be a relevant driver of its habitat use as seen in other species of small wild felids [27–29]. We used camera traps in conjunction with occupancy and cooccurrence modeling to determine how the habitat use of Andean tiger cats is modulated by different habitat conditions and by potential competitors, intraguild killers/predators and prey. To answer these questions, we surveyed three protected cloud forests comprising a key location in the Middle Cauca River Basin (hereafter Middle Cauca) in Colombia.

### Hypotheses

Increased survey effort has been linked to higher detectability of other neotropical small wild felids [5, 30]. Detectability may also vary among camera brands due to differences in the camera features, such as the time delay of the passive infrared (PIR) sensor [31]. It has been suggested that the use of cameras with shorter PIR time delays increases the probability of detecting fast-movement animals [22]. Thus, we expected that Andean tiger cat detectability would increase with a greater sampling effort [5] and shorter PIR time delays.

The structural complexity of the habitat is the degree of both horizontal and vertical spatial heterogeneity present in the forest structure [32]. We expected that the habitat use of Andean tiger cats would increase in sites with more complexity in the vertical and horizontal forest structure dimensions since they represent greater availability of prey and refuge [4, 6]. We also expected Andean tiger cat habitat use and detectability to decrease in sites with a steeper slope given its related energetic investment in movement [33, 34].

Since they are forest-dwelling felids, we expected that Andean tiger cats would negatively respond to forest fragmentation and landscape heterogeneity but positively respond to forest amount and patch cohesion [6, 16]. We expected that a greater human footprint legacy in the study areas would negatively affect the habitat use of Andean tiger cats, as has been previously found for other neotropical mammals [35]. Proximity to human settlements and roads relates to detrimental activities for Andean tiger cats, such as unregulated tourism, roadkill, illegal hunting, and encounters with dogs, thus, we expected greater habitat use for the species in sites distant from these features [20, 36].

Small mammals are a pivotal energy source for tiger cats, representing nearly half the biomass of their prey [2]. We expected that Andean tiger cats would use sites with greater small mammal occupancy to increase hunting success [3, 27]. We also expected that Andean tiger cats would avoid sites where other carnivores of greater body mass might be present to reduce potential encounters that could promote intraguild killing or competition [37]. Theory predicts that in cases where resources are abundant (*e*.*g*., prey), dominant carnivores tend to exclude subordinate carnivores, whereas if resources are scarce, the dominant carnivore is excluded, but the subordinate thrives through specific adaptations [10].

## Materials and methods

### Ethics statement

We used noninvasive methods for data gathering, and hence, approval from institutional or animal ethics committees was not needed. Our field team was verbally authorized by the local communities and specific landowners to survey the study areas.

### Study area

The camera trap survey was conducted in three different protected areas from the Middle Cauca, which is a river basin located between the Western and Central Cordilleras in the Northern Andes of Colombia. The Mesenia-Paramillo Nature Reserve (MPNR) and Cuchilla del San Juan Integrated Management District (CSJIMD) are located on the eastern slope of the Western Cordillera (Fig 1B, 1C). The Campoalegre Soil Conservation District (CSCD) is located on the western slope of the Central Cordillera (Fig 1D) and is the largest reserve surveyed with an area of 211.3 km^2^, followed by CSJIMD with 117.9 km^2^ and MPNR with 35 km^2^. The average annual temperature in the study areas ranges from approximately 15–23°C, and the average annual rainfall is 1,900 to 3,100 mm [41]. The most representative native vegetation cover in the region is the northwestern Andean cloud forest. These forests present stunted and gnarled canopies with high cover of epiphytes and an understory permanently covered by abundant leaf litter, adventitious roots, and fallen trunks [42]. In the surrounding zones of the protected areas, it is common to find large expanses of pastures for cattle ranching, eucalyptus and pine plantations, and abandoned plots with secondary vegetation and crops (mainly coffee and avocado) [15, 43]. There is unmanaged tourism as well as illegal logging and hunting both within and around CSCD and CSJIMD, with both protected areas facing a lack of personnel to control this kind of human activity. In contrast, the MPNR is the only protected area that has trained forest rangers that regulate tourist entry and prevent hunting and logging within its limits. Consequently, the MPNR has a greater proportion of cloud forests in contrast to other existing covers in the studied landscape (80%) compared to CSJIMD (67%) and CSCD (66%), which are currently under expansion due to ongoing restoration efforts.

### Sampling design

We set 21 camera traps in MPNR, 22 in CSJIMD and 23 in CSCD inside their native forest covers, which were arranged in a 1 km^2^ grid with an average spacing of 962 ± 84 m following previous study designs on space use by neotropical small felids [30, 37, 44, 45]. Excluding the damaged or stolen cameras, we were able to collect data from 58 sampling stations for subsequent analysis (Table 1). Prior to installation, we removed the vegetation within a strip in front of the camera’s detection field that was 1 m wide and 5 m long to avoid ‘ghost photos’ caused by wind-blown vegetation [46]. At each station, a single camera was attached at 30 cm high along natural trails favored by felids, without the use of lures [36]. We used several brands and models of passive infrared camera traps for each protected area (APEMAN ® Trail Cam H45, Blaze Video ® A262 Animal Cam, Browning ® Strike Force Apex and Strike Force Gen 5, Bushnell ® Trophy Cam HD, Trophy Cam 119717CW, Trophy Cam Agressor, Reconyx ® Hyperfire, and Victure ® HC300). We set the cameras to take sequences of three photos with the minimum delay possible (0–5 s depending on the brand). The sampling effort and the number of cameras varied between reserves (Table 1), and the average sampling duration for each camera location was 130 ± 24 days. The MPNR sampling period was from August 2020 to February 2022, the CSJIMD was from December 2021 to June 2022, and the CSCD was from February to November 2021.

**Table 1:** Protected area, sampling area, number of active stations (excluding stolen or damaged cameras), elevation range covered by the camera grid, and number of independent detections of the Andean tiger cat, tayras, intraguild killers/predators, and prey.

PA	MCP (km^2^)	Active stations	Elevation range (m asl)	Effort (trap/nights)	NID (NID per 7-day occasion)			
					Andean tiger cat	Tayra	Intraguild killers/predators	Prey
MPNR	26.57	19	1,700–3,177	4,518	109(70)	89(63)	54(46)	532(184)
CSJIMD	26.49	19	2,200–3,200	2,829	56(46)	52(41)	17(16)	215(119)
CSCD	21.02	20	1,800–3,800	3,342	16(13)	17(14)	12(11)	218(120)

Abbreviations: PA; protected area, MCP; minimum convex polygon of the camera trap array, MPNR; Mesenia-Paramillo Nature Reserve, CSCD; Campoalegre Soil Conservation District, CSJIMD; Cuchilla de San Juan Integrated Management District, NID; number of independent detections.

#### Covariates

We included covariates on the microhabitat and landscape scales based on *a priori* biological hypotheses of their effects on the habitat use of Andean tiger cats (S1 Table). We quantified the number of days in which the cameras were active to estimate the potential effects of the survey effort on Andean tiger cat detectability [47]. To minimize the biases associated with the variation in the camera trap functionality, we included the minimum PIR time delay of each brand as a categorical covariate conditioning detectability [48].

#### Landscape scale

We describe landscape structure using a 30 m resolution land cover classification product that fixes the error induced by cloudiness in the study area [38]. We created buffer areas with a 500 m radius using the location of each camera trap as the area centroid to quantify class-derived landscape metrics (S1 Table). Even though 500 m seems to be a small buffer radius for making landscape-scale inferences in the occupancy of small wild felids under some environmental contexts [37]. The area defined by this buffer is projected in a two-dimensional instead of a three-dimensional surface. This issue is usually ignored in studies involving mobile organisms immersed in landscapes with vertical relief [34]. In particular, felids may have greater constraints on their habitat use in three-dimensional (*e*.*g*., valleys and hills in the highland cloud forests) rather than two-dimensional (*e*.*g*., plains and lowland rainforests) environments, as the former have a greater surface area [49]. Thus, according to the broken topography inside the 500 m buffer of each site surveyed, the minimum home range size (0.9 km^2^) reported for lowland members of the *tigrinus* complex [37] and recent evidence on the occupancy of other small wild felids elsewhere [6, 50, 51]. We might expect that a 500 m buffer would be an accurate spatial scale for modeling the effects of landscape predictors on the habitat use of the Andean tiger cat, at least in our study system.

We estimated four landscape metrics reflecting landscape heterogeneity (Shannon’s index), forest amount, edge density and forest patch cohesion index using the LecoS plugin of QGIS 3.16 software [52]. We constructed a Pearson’s correlation matrix to inspect for collinearity issues (r > |0.65|) among landscape structure covariates. We noted that forest amount showed a strong positive correlation with patch cohesion index (r = 0.83) and a strong negative correlation with landscape heterogeneity (r = -0.92) and edge density (r = -0.86). Given the correlation structure we found in our landscape metrics, we decided to summarize their effects with a single landscape structure covariate (LS) after applying principal component analysis [16]. According to Kaiser-Kupferman’s criterion, the most important linear combination was determined using the first principal component, as it presented eigenvalues > 1 [4, 16, 53]. The negative values of the first principal component represented a positive gradient of amount and cohesion of forest patches, while the positive values reflected greater habitat heterogeneity and forest edge density. Further details on this covariate are provided in the supplementary material (S1 Fig).

To determine whether the habitat use of Andean tiger cats increased monotonically with increasing elevation or if it decreased when elevation peaked value, we also tested the linear and quadratic effects of elevation [35]. The Legacy Human Footprint Index (LHFI) combines the effects of land use intensity and time to intervention in the landscape at a resolution of 300 m and we obtained the average value for each station with 500 m buffers [21]. From cartographic information provided by the Departamento Administrativo Nacional de Estadistica – DANE GeoPortal (https://geoportal.dane.gov.co/), we calculate the Euclidean distance between each sampling station and the nearest roads and human settlements using the QGIS 3.16 plugin NNJoin.

#### Site-scale

As an indicator of understory vertical complexity, leaf litter depth was determined using a measuring tape and a rod that was slowly driven into the soil at a 90° angle until it reached the rhizosphere [54, 55]. To quantify the horizontal microhabitat structure, we used a 50 x 50 cm wooden square composed of a nylon net with 100 grids to measure the proportion of canopy, herbaceous, and leaf litter cover at each station [56]. Canopy height is a covariate that often represents the vertical complexity in the canopy [57], and we measured it with a Nikon Prostaff 550 laser. We measured the slope with a Suunto PM-5/360 PC clinometer as an indicator of the microhabitat topographic inclination. For each sampling station, we recorded measurements at the point where the camera was installed and at 5 m in each cardinal direction [6]. We averaged the measurements obtained for each microhabitat covariate and used the resulting value as input for subsequent modeling [6].

We scaled all the numerical covariates to facilitate direct comparison between model estimates and improve parameter estimation through the following formula: raw data - average/standard deviation [47, 58]. Subsequently, to minimize bias in the modeling, we removed numerical covariates with multicollinearity or uniformity problems (S2 Fig), which were detected via graphical inspection and a correlation Pearson coefficient [4, 47]. We found a strong positive correlation between elevation and distance to roads, as well as a strong negative correlation between herbaceous cover and leaf litter cover and between LHFI and distance to human settlements (S2 Fig). The final set of covariates used in the occupancy models for the Andean tiger cat included landscape structure, protected area, canopy height, canopy cover, leaf litter cover, leaf litter depth, slope, distance to human settlements and elevation. For the modeling of detection probability, the final set of covariates included herbaceous cover, slope, detection delay of the passive infrared sensor and survey effort of the camera traps.

### Occupancy modeling

We considered the occasions to be independent and pooled the pictures of each station over a 24 h period. For the design of the detection/nondetection histories, we collapsed the survey into weekly periods to reduce zero-inflation in the matrix and improve model convergence [35]. In the occupancy modeling, we defined each camera-trap station as the sampling unit [23]. Since the movement patterns of the target species are larger relative to the area covered by the detection field of our sampling units, we interpreted the results of our occupancy and detection estimates as habitat use and intensity of use, respectively [23].

To test the relationships between the habitat use of the Andean tiger cat and the selected covariates, we built single-species-single-season occupancy models, establishing two hierarchically linked processes: ecological and observational. The first represents the occupancy probability (ψ_ij_), defined as the probability of the species occurring or using a site during a sampling season [23]. This parameter is represented as a latent variable with Bernoulli distribution and descriptor function of the form z_i_∼Bernoulli (ψ), where Ζ is the actual occupancy value at each site i with probability of occurrence ψ [59]. The observation process represents the imperfect detection of the species and is represented by the probability function p_ij_ |Ζ_i_∼Bernoulli(ψ_i_*p_ij_), where p_ij_ is the observation at each site i at each visit j if site i is occupied [23]. These parameters can be modeled as a function of covariates in a logistic regression of the form logit (ψ_i_) = β_0_ + β_1_x_i0_ for occupancy or logit (p_i_) = α_0_ + α_1_x_i_ for detection [59], where β_0_ and α_0_ are intercept values from which the model starts and x_i_ is the covariate(s) measured for each station i.

To determine the most influential covariates affecting the occupancy of Andean tiger cats, we used a Bayesian modeling framework [60]. We fit each model with uninformative priors and ran three Markov Chain Monte Carlo (MCMC) samplers of 30,000 iterations, from which the first half was discarded as burn-in. Additionally, we calibrated the models with an acceptance sample (adapt_delta) of 0.99 for the adaptation period of the MCMC sampling [61]. A value of 0.99 for adapt_delta means that the algorithm will explore the parameter space more thoroughly during adaptation, which can lead to better quality posterior samples [62]. We used the expected log pointwise predictive density (elpd) to compare and rank the models, as it is a measure of model predictive accuracy [58]. The elpd reflects how the model predicts future data, assuming that it came from the same distribution as the observed data [63]. To assess model support relative to the top model, we also computed the pairwise differences in elpd (Δelpd) between models and the top model. We performed model selection using the leaving one out (LOOIC) cross-validation information criterion on the estimates of the posterior distribution [63]. LOOIC removes one data point at a time from the dataset, fits the model to the remaining data, and then uses the model to predict the value of the removed data point. This process is repeated for each data point to obtain an estimate of the model’s predictive accuracy [64]. We considered a model to have less support than the top model if its absolute difference in elpd was greater than the standard error of that difference [63]. We then performed sequential-by-selection submodeling using the p-first approach [65]. In the first stage, we modeled the detection process by fixing the individual effect of its associated covariates while keeping ψ constant [35]. In the second stage, we modeled ψ by individually fixing its corresponding covariates while fixing the model for p with its most relevant covariate, as previously determined [65].

To test for unaccounted variation in the relationship between the selected covariates at each study location (*e*.*g*., MPNR, CSCD, and CSJIMD) and the occupancy of Andean tiger cats, we included the former as a random effect in the same modeling structure. However, this model did not perform better than models without the random effect (S2 Table). Recent evidence suggests that, when models include a random effect with fewer than five factorial levels, it is convenient to return to a model with fixed effects to prevent inflated variances [66]. We inferred the influence of the supported covariates on the top-ranked occupancy and detection models by inspecting the β coefficients and their 95% credibility intervals [60]. We looked for positive or negative trends in the relevant covariates of each model by determining if the credibility intervals had non-zero overlap and positive or negative values, respectively [4]. We assessed model convergence by inspecting the Gelman-Rubin Statistic (R-hat); R-hat<1.1 indicates that the models are acceptable [67]. We tested the models for overdispersion via a posterior predictive check using the MacKenzie-Bailey test for the most parsimonious models [23]. We computed this test for the real data and for the simulated dataset in each iteration of the MCMC sampling [60]. If the model showed a good fit, the proportion of draws in which the simulated estimator is greater than the real estimator should be greater than 0.05 (Bayesian P value > 0.05) [59, 60]. Bayesian occupancy models were built using the *ubms* package [60] version 1.1.0 in R software. To assess spatial autocorrelation, we applied Moran’s I index to the residual component of the top-ranked models for the occupancy of the Andean tiger cat and visually inspected spline correlograms for correlation patterns across the distance unit [68]. We obtained the model residuals for the occupancy component defined by Wright et al. [69]. The spatial autocorrelation analysis was performed using the *nfc* package [70] version 1. 3. 0.

### Cooccurrence modeling

To model the effect of the occupancy and detection of other species on the occupancy and detection of Andean tiger cats, we implemented conditional cooccurrence models [71] using the ΨBa parameterization (Table 2). This is an asymmetric scenario established between two interacting species, where the occupancy and detectability of one species is assumed to be conditional on the occupancy and detectability of another species given some *a priori* criteria [10, 71]. To assess whether Andean tiger cat occupancy and detection were related to that of prey, we assigned all detected small mammals as the dominant species (ΨA) and the Andean tiger cat as the subordinate species (ΨB). In the context of the spatial cooccurrence between Andean tiger cats and sympatric carnivores, we designated the former as the subordinate (ΨB) due to its small body size, restricted geographic range, and previous evidence of competitive exclusion with sympatric felids [44, 72].

**Table 2:** Parameters in the conditional cooccurrence modeling of the Andean tiger cat with its prey, intraguild competitors and killers/predators in three protected cloud forests from the Middle Cauca, Colombia.

Parameter	Description
ΨA	Occupancy probability of intraguild competitors, killers/predators, and prey
ΨBA	Occupancy probability of Andean tiger cats given intraguild competitors, killers/predators, and prey presence.
ΨBa	Occupancy probability of Andean tiger cats given intraguild competitors, killers/predators, and prey absence
pA	Detection probability of intraguild competitors, killers/predators, and prey given Andean tiger cat absence.
pB	Detection probability of Andean tiger cats given the absence of intraguild competitors, killers/predators, and prey
rA	Detection probability of intraguild competitors, killers/predators, and prey given the presence of both species.
rBA	Detection probability of Andean tiger cats given the presence of both species and the detection of intraguild competitors, killers/predators, and prey
rBa	Detection probability of Andean tiger cats given the presence of both species and the nondetection of intraguild competitors, killers/predators, and prey

The dominant intraguild members were identified according to body mass differences, trophic overlap, and the sample size obtained to improve parameter estimation of the data-hungry cooccurrence models [67, 72–74]. The cooccurrence of Andean tiger cats against other potentially dominant intraguild members was analyzed in two different ways. First, we analyzed the cooccurrence of Andean tiger cats conditional on tayras as potential intraguild competitors. In some areas, tayras exhibited high dietary overlap with tiger cats and similar body mass ratios [9, 74] and, in our context, had an appropriate sample size compared to any other intraguild species to model conditional parameters. Second, we analyzed the cooccurrence of Andean tiger cats conditional on a category of potential intraguild killers/predators (*e*.*g*., pumas, ocelots, dogs, and Andean bears). Overall, the selected species exceed by far the body mass ratio required to induce a safety kill over an Andean tiger cat [9] but where combined together due to their low sample size to improve model convergence. We recognize that this coarse taxonomic resolution hinders us from discriminating fine-scale differences in the intensity of the cooccurrence outcome for intraguild killers/predators. Nevertheless, the selected species have the potential to limit the occupancy of Andean tiger cats either by direct predation or killing to avoid encounters during the use of a particular resource [37, 74]. Therefore, irrespective of the kind of antagonism, we might expect a nonrandom response in the use of a given site by Andean tiger cats as a function of the presence of other carnivore species.

Once we defined the conditional state of each taxon, we built models assuming that the occupancy of ΨB was (ΨBA ≠ ΨBa) or was not (ΨBA = ΨBa) affected by the presence of ΨA. Furthermore, we modeled all detection parameters within both independent (pA = rA, pB = rBA = rBa) and dependent (pA ≠ rA, pB ≠ rBA ≠ rBa) cooccurrence states. We performed model selection based on the Akaike information criterion (AIC) corrected for small samples (AICc), and the best models were retained according to a conventional parsimony threshold (ΔAICc < 2) [75].

As with occupancy models, transformed β coefficients were used to infer either positive or negative relationships between covariates and conditional parameters [4]. Covariates from the best single occupancy models were included in the cooccurrence models to account for habitat preferences in imperfect detection. If the best models retained a conditional parameter structure (ΨBA ≠ ΨBa or rBA ≠ rBa), we estimated the species interaction factor (SIF) and species detection factor (DIF). The SIF (Φ) represents the probability that two species of interest cooccur nonrandomly in the landscape and is calculated as follows: Φ = (ΨA ΨBA)/ΨA (ΨA ΨBA + (1- ΨA) ΨBa [76]. If φ > 1, the two species tend to coexist more than expected by chance; if φ < 1, the species tend to evade one another; and if φ = 1, the species exist independently, and there is no evident pattern of evasion or aggregation [71]. The DIF (δ) represents the codetectability, in other words, the probability of two species being nonrandomly codetected, and is calculated as follows: δ = rBA/(rA rBa). If δ ≠ 1, then the detection probabilities of both ΨB and ΨA are conditional on the other, as in the SIF. Conditional cooccurrence models were built using the *wiqid* package [77] version 0. 3. 3. through R Software.

## Results

After 10,689 trap/nights in a sampling area of 74.1 km^2^, we obtained 129 independent detections of Andean tiger cats under a 7-day occasion framework at 38 sites (54% - MPNR, 36% - CSJIMD, 10% - CSCD), 118 independent detections of tayras at 39 sites, 73 independent detections of potential intraguild killers/predators at 27 sites (61% - MPNR, 27% - CSJIMD, 12% - CSCD) and 463 independent detections of prey at 49 sites (55% - MPNR, 22% - CSJIMD, 23% - CSCD). Of the records obtained for intraguild killers/predators, 33% corresponded to Andean bears (9 sites), 27% to dogs (9 sites), 26% to pumas (11 sites), and the remaining 14% to ocelots (8 sites). We found that 77%, 76%, and 72% of the sites with Andean tiger cat presence also manifested presence of tayras, prey, and potential intraguild killers/predators, respectively.

### Occupancy modeling

Hereafter, we present the results obtained for the three protected areas together, yet separate estimates can be found in the supplementary material (S2 Table). The null model showed that there was a 15% chance of obtaining a weekly detection of the Andean tiger cat if present (p = 0.15, SD = 0.02, CI = 0.12–0.17) and an average occupancy of 68% (Ψ = 0.68, SD = 0.06, CI = 0.55–0.80). The top-ranked model showed that there was 13% chance of obtaining a weekly detection of the Andean tiger cat if present (p = 0.13, SD = 0.02, CI = 0.09–0.18) and an average occupancy of 73% (Ψ = 0.73, SD = 0.08, CI = 0.56–0.88).

**Fig 2:**
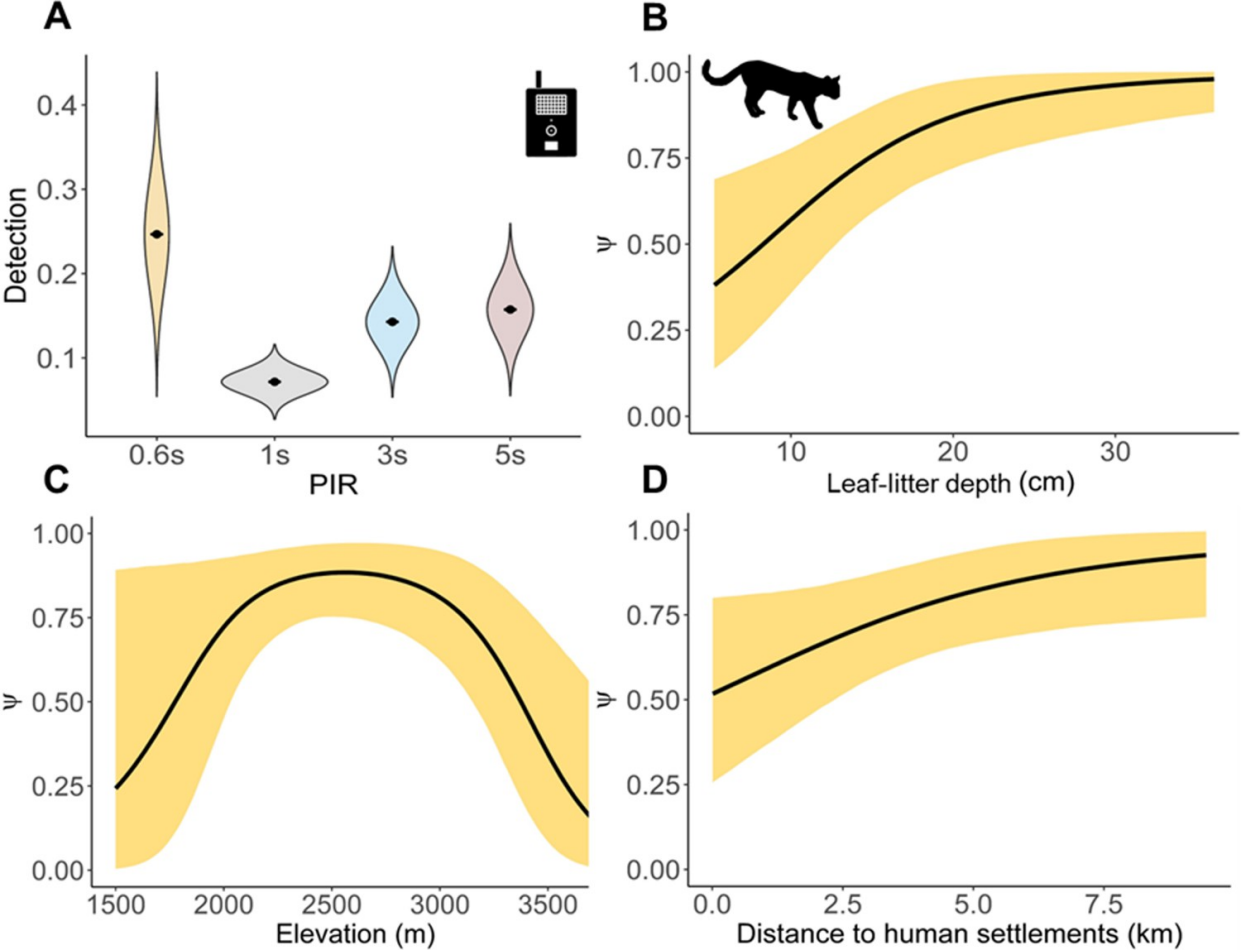
Predictions of the posterior distribution for the occupancy and detection of Andean tiger cats in three protected cloud forests from the Middle Cauca. The detection of the Andean tiger cat was predicted against the time delay in seconds (A) of the passive infrared sensor. The occupancy of the Andean tiger cat was predicted against the covariates representing (B) leaf litter depth, (C) quadratic elevation, and (D) distance to human settlements. The orange shading represents the 95% credibility intervals. PIR is defined as the passive infrared sensor of the camera trap brands.

The top-ranked model for Andean tiger cat detection included the effects of the camera trap’s PIR time delay (Table 3). Detection was higher for the species at sites that had cameras with a PIR time delay ≤ 0.6 seconds (Fig 2A). The top-ranked models for Andean tiger cat occupancy included the effect of leaf litter depth, quadratic elevation, and distance to human settlements (Table 3). Leaf litter depth had a positive and marked effect on the occupancy of Andean tiger cats (β = 1.38, SD = 0.55, CI = 0.41–2.57, R-hat = 1) (Fig 2B). The occupancy of Andean tiger cats reached a maximum between 2,000 and 3,000 m asl, rapidly decreasing above or below this threshold (β = -0.99, SD = 0.41, CI = -1.85 – -0.21, R-hat = 1) (Fig 2C). Distance to human settlements had a positive but weak effect on the occupancy of Andean tiger cats (β = 0.81, CI = 0.0372–1.71, R-hat = 1) (Fig 2D). Goodness-of-fit tests performed to identify the best models showed a marginal fit and signs of underdispersion (S3 Fig). Moreover, we did not find any sign of spatial autocorrelation in Andean tiger cat occupancy (S4 Fig).

**Table 3:** Estimates of the posterior distribution of the occupancy and detection parameters obtained for Andean tiger cats in three protected cloud forests from the Middle Cauca, Colombia.

Parameter	Model	ELPD	K	ELPD_Δ_	SE ELPD	ELPD weight
P	p(PIR)Ψ(.)	-392.936	11.316	0	0	0.654
	p(.)Ψ(.)	-396.19	3.731	-3.254	6.391	0.025
	p(SL)Ψ(.)	-397.562	6.007	-4.626	6.522	0
	p(CF)Ψ(.)	-398.459	8.515	-5.523	6.796	0.145
	p(E)Ψ(.)	-399.034	8.693	-6.098	7.407	0.177
Ψ	p(PIR)Ψ(PH)	-388.967	11.825	0	0	0.442
	p(PIR)Ψ(Elevation^2^)	-389.842	13.068	-0.875	2.735	0.216
	p(PIR)Ψ(PA)	-391.324	12.551	-2.357	3.009	0
	p(PIR)Ψ(HUM)	-391.347	11.835	-2.38	3.43	0.071
	p(PIR)Ψ(CH)	-392.521	12.539	-3.554	2.535	0
	p(PIR)Ψ(Elevation)	-393.143	13.076	-4.176	3.175	0
	p(PIR)Ψ(CD)	-393.523	12.952	-4.556	2.969	0
	p(PIR)Ψ(SL)	-393.581	11.891	-4.614	2.566	0
	p(PIR)Ψ(AD)	-393.906	12.567	-4.939	2.918	0
	p(PIR)Ψ(LS)	-393.959	12.778	-4.992	2.424	0
	p(.)Ψ(.)	-396.19	3.731	-7.223	6.589	0.271

Abbreviations: ELPD; expected predictive accuracy, K; number of parameters, ELPD_Δ_; difference in expected predictive accuracy between any model and the best model, SE; standard error of the difference in predictive accuracy, PIR; passive infrared sensor, SL; slope, CF; herbaceous cover, E; effort, PH; leaf litter depth, PA; protected area, HUM; distance to human settlements, CH; leaf litter cover, CD; canopy cover, AD; canopy height, LS; landscape structure.

### Cooccurrence modeling

**Fig 3:**
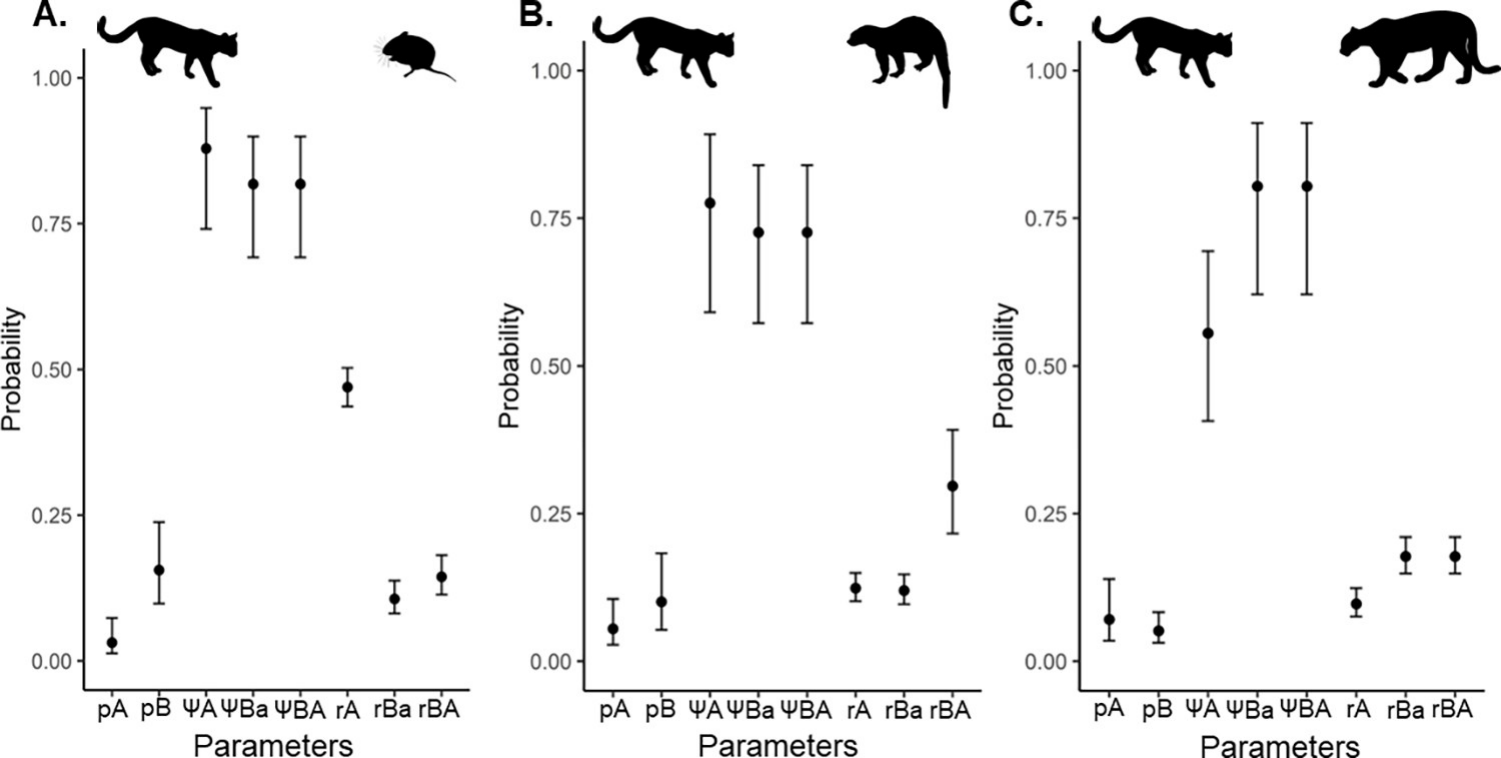
Coefficients of the parameters estimated by the conditional cooccurrence models of Andean tiger cats against (A) potential prey, (B) tayras, and (C) potential intraguild killers/predators. Vertical bars represent 95% confidence intervals. Parameter abbreviations: pA; detection probability of intraguild competitors/killers/predators and prey given Andean tiger cat absence, pB; detection probability of Andean tiger cats given the absence of intraguild competitors, killers/predators and prey, ΨA; occupancy probability of intraguild competitors, killers/predators and prey, ΨBa; occupancy probability of Andean tiger cats given intraguild competitors, killers/predators and prey absence, ΨBA; occupancy probability of Andean tiger cats given intraguild competitors, killers/predators and prey presence, rA; detection probability of intraguild competitors, killers/predators and prey given the presence of both species, rBa; detection probability of Andean tiger cats given the presence of both species and the nondetection of intraguild competitors, killers/predators and prey, and rBA; detection probability of Andean tiger cats given the presence of both species and the detection of intraguild competitors, killers/predators and prey.

The cooccurrence modeling procedure revealed a random cooccurrence pattern of Andean tiger cats with their prey, tayras and intraguild killers/predators (S3 Table). However, the Andean tiger cat detection probability was higher when prey was present and detected (rBA = 0.14, CI = 0.11–0.18) than when it was undetected (rBa = 0.10, CI = 0.08–0.13). This was also the case when comparing the Andean tiger cat detection probability with that of intraguild competitors (Fig 3). Andean tiger cat detectability was lower when tayras were present but undetected (rBa = 0.11, CI = 0.09–0.14) than when they were present and detected (tayras: rBA = 0.29, CI = 0.21–0.39). In addition, there was no evidence to support that Andean tiger cat occupancy or detectability was conditional on that of its intraguild killers/predators (S3 Table). However, the detectability of intraguild killers/predators increased at sites with presence of both Andean tiger cats and intraguild killers/predators (rA = 0.09, CI = 0.07–0.12), compared to sites where only intraguild killers/predators were present (pA = 0.07, CI =0.03–0.13).

The SIF was 1.0 when comparing the Andean tiger cat occupancy conditional on the presence of either prey, tayras, or intraguild killers/predators, suggesting that Andean tiger cats are using the sites regardless of their presence. Nevertheless, the DIF was positive when comparing Andean tiger cat detection conditional on the detection of prey (δ = 1.41), and tayras (δ = 3.10). None of the relevant covariates for predicting the occupancy of Andean tiger cats could explain the patterns of cooccurrence with their prey, tayras, and killers/predators (S3 Table).

## Discussion

This is the first detailed study assessing the habitat requirements for Andean tiger cats in highland ecosystems across its geographic range. The habitat use of the Andean tiger cat was mainly affected by leaf litter depth, elevation, and, to a lesser extent, the distance from human settlements. We did not find support for conditional cooccurrence between Andean tiger cats and either prey or intraguild competitors and killers/predators, but we did find that their detectability increased when both prey and tayras were present and detected. Overall, we found that Andean tiger cats occurred in more than half of the sampling units in the studied protected areas but were imperfectly detected in the surveys. Our results agreed with some previous estimates of occupancy and detection for the tiger cat species complex from the cloud forests of the Western Cordillera to the Atlantic Forest and the Caatinga drylands (S4 Table).

The covariate most relevant for the Andean tiger cat detectability was the PIR time delay, with PIR = 0.6 being the shortest found in the camera brands used. This finding aligns with both experimental and empirical evidence, which suggests that camera brands with short time delays are optimal to detect carnivores, as they tend to be fast-moving species [48, 67]. Thus, we suggest using faster PIR camera brands in future surveys to improve Andean tiger cat detectability. Contrary to the trends observed for other neotropical small felids, we found that the camera trap effort showed no relevant effect on the detectability of the Andean tiger cat [5, 30, 61].

We found a nonlinear response of Andean tiger cats to elevation and a greater use of sites located at approximately 2,000 to 3,000 m asl. Elevation was previously reported as an influential driver of habitat suitability for Andean tiger cats in Colombia, with more suitable habitats at higher elevations [78]. We observed a greater use of sites at middle elevations, which could be related to prey availability likely represented by the assemblages of small mammals [79, 80]. In Andean cloud forests, the diversity of small mammals tends to be greater at middle elevations, as there is greater species turnover and productivity [81]. Moreover, we found a striking correlation of the sampling sites more distant to roads with high elevations, which may also affect the habitat use of the Andean tiger cat. In the Northern Andes of Colombia, areas at high elevations are less accessible than those at low elevations which might reduce the pressures related to encounters with humans, domestic animals, or vehicles [21, 82]. The constrained access to forested areas at higher elevations may also discourage forest loss while improving habitat conditions for small felids, as has been found in mountain forests of Sumatra [83].

Meanwhile, climate change could affect range-restricted species at middle elevations because Andean ecosystems are moving to higher elevations as an effect of increased environmental warming [84, 85]. Under environmental warming, cloud forests are expected to suffer an altitudinal attrition of their cloud formations, which will compromise the integrity of their bottom-up processes [85]. As a consequence, species with restricted niches will be more susceptible to range shifts and contractions due to resource depletion and increased competition against migrant species from the lowlands [85]. Notably, by the 2050s, climatic forecasts under the “business as usual scenario” indicate that the current members of the *tigrinus* complex will suffer the greatest range contraction of any neotropical small felid, losing 86.2% of its actual range [86]. Regardless, the authors suggest that the conflictive taxonomy of the *tigrinus* complex could prevent any conclusion on their imminent risks and urge a fair diagnosis between the highland and lowland forms [86]. More research is needed to confirm whether the geographic range of the Andean tiger cat would suffer a similar or different fate due to the warming of the inter-Andean valleys from northwestern Colombia.

Contrary to our expectations, the landscape structure aspects we measured within the protected areas did not influence Andean tiger cat habitat use. It has been suggested that greater landscape heterogeneity increases prey availability and, consequently, the habitat use of small wild felids [87, 88]. However, the flexible use of disturbed areas by these organisms depends on the landscape context since the availability of conserved neighboring habitats is required [16]. Our results aligned with a study conducted in the highlands of Ecuador, which found no effects of habitat loss or fragmentation on the habitat use of pumas, Andean bears, Andean foxes (*Lycalopex culpaeus*), and strip hog-nosed skunks (*Conepatus semistriatus*). This study suggested that, regardless of anthropogenic landscape transformation, the high Andes ecosystems are already heterogeneous, comprising forests, shrubs, and grasslands (paramos), which could explain the widespread carnivore occurrence in this landscape [89]. Nonetheless, plasticity in trophic preferences may enable some species to adapt more easily than others in heterogeneous landscapes [16]. For example, omnivorous species (*e*.*g*., foxes, Andean bears) are able to use more resources than strict carnivorous species, which is favorable in terms of increased permeability to the existing conditions in the matrix [16].

We found a positive effect between the habitat use of Andean tiger cats and the distance to human settlements, which was consistent with the trends found for lowland tiger cat populations [90]. However, in Colombia, Andean tiger cats have been reported near human settlements and even in peri-urban areas in major cities [78, 91–93]. These observations are commonly based on a few indirect or bycatch records of camera traps and tracks, with no explicit information relating Andean tiger cat populations with proximity to urban areas. Accounting for imperfect detection, we confirmed that Andean tiger cats negatively respond to human settlements, at least for our study sites, which belonged to a central portion of one of the highest populated and economically active regions in Colombia, the Middle Cauca. Therefore, any prior suggestions of Andean tiger cat tolerance to occupy more anthropized environments should be interpreted with caution. A key issue is that our models were based on the overall conditions of the three protected areas sampled, so we suggest precluding any extrapolation of these results to other locations with different conditions, either inside or outside the region. There is evidence suggesting that near human settlements, the mortality risk for tiger cats increases due to interactions with humans in the form of vehicle collisions [20], retaliation in poultry depredation and illegal fur trade [17]. Close to human settlements, light and noise pollution may also affect the space use of wild felids by introducing fear of encounters with humans [94].

We found a strong positive correlation between the sampling sites closer to human settlements and the LHFI. Cloud forests of the middle Cauca proximal to human settlements are known to be differentiated from more distant forests by having a more intense land use change and a longer history of interventions [82]. In particular, the economic balance of industries such as cattle, coffee, and avocado farming relies mainly on the presence of roads and the proximity to intermediate and large population zones [95]. This suggests that Andean tiger cats are negatively impacted by other synergic anthropogenic factors not considered here that may prevail in the region, which also requires expanding our current modeling framework. The occupancy of other small felids in South America has been found to decrease with a greater abundance of livestock and dogs [29, 50]. Including the individual effect of such factors as potential drivers of Andean tiger cat habitat use would improve our discernment of its response to human disturbance.

We found that Andean tiger cat habitat use was positively related to leaf litter depth, which is generally indicative of a mature healthy cloud forest. Mature cloud forest understories have abundant leaf litter and prominent adventitious roots coupled with slow decomposition rates and low nutrient concentrations, respectively [81]. The vertical structural complexity of this microhabitat may promote the habitat use of Andean tiger cats by providing them food and shelter [96]. Deep leaf litter can provide an ideal ambush site, as small mammals tend to seek these conditions for thermoregulation, feeding, or nesting [55, 96]. A recent study showed that leaf litter depth had a strong positive effect on the habitat use of two sympatric species of short-tailed opossums (*Monodelphis* spp.) in the eastern Amazon [55]. Deep leaf litter was also positively related to the abundance of three terrestrial (*Thomasomys laniger*, *Neomicroxus bogotensis*, *Cryptotis cf*. *tamensis*), and two arboreal (*Marmosops caucae*, *Rhipidomys fulviventer*) small nonvolant mammals in an oak forest in the Eastern Andes of Colombia [96]. We also postulate that the vertical complexity in the understory may provide cavities and galleries of various sizes that can be used by Andean tiger cats and other small carnivores to avoid intraguild killing. Slope showed no effect on Andean tiger cat habitat use and detectability. Carnivores such as Andean bears and pumas tend to prefer sites with little slope, such as ridges or streams, to facilitate their movement [97, 98]. We postulate that Andean tiger cats do not deviate from this overall pattern of carnivore habitat use in rugged terrain.

Conditional cooccurrence models showed that Andean tiger cat habitat use was not affected by the presence of prey, but Andean tiger cat detectability increased when prey was present and detected. In other words, Andean tiger cats used areas that had more prey more frequently. A plausible explanation for this pattern is that Andean tiger cats use sites with greater intensity of use by prey to increase hunting encounters, which is expected for an opportunistic predator [3]. In temperate ecosystems, small carnivores face a metabolic trade-off between maximizing food intake and avoiding heat loss, as environmental temperatures tend to reach extremely low limits [99]. Preliminary evidence on the activity patterns of Andean tiger cats in the surveyed localities also supported this relationship, since there was a marked overlap between Andean tiger cat and small mammal activity.

No support for spatial segregation was found in the habitat use of Andean tiger cats regarding the presence or absence of tayras and potential intraguild killers/predators. Nevertheless, the abundance of the cat’s *de facto* potential killers/predators was low and hence would not be expected to lead to meaningful behavioral changes [36, 100]. Tayras, the most abundant sympatric carnivore, exhibited, in fact, a greater likelihood of being a competitor rather than an actual killer of Andean tiger cats, as they showed high dietary overlap with small mammals but fell below the minimum body mass required for a “safety” intraguild killing [9, 74]. Thus, it is expected that habitat structure and temporal segregation would act as the modulating factors driving coexistence in this highland environment, as intraguild killers/predators are rare and potential competitors are mostly diurnal and do not depend only on meat to survive. Additionally, being highly territorial, felids use a complex system of scent marking with their urine and feces to delimit areas of exclusive and common use [101]. Hence, we postulate that Andean tiger cats could evade confrontation by recognizing areas recently marked by other sympatric carnivores, but this hypothesis needs to be further explored.

In lowland neotropical forests, ocelots tend to suppress populations of smaller mesocarnivore species, such as tiger cats, through the ocelot/*pardalis* effect [37, 100]. When ocelot populations reach numbers above a certain threshold, small wild felids decline either due to intraguild killing or competitive interference [27, 36, 37, 100]. Nonetheless, we suggest that in cloud forests from the Middle Cauca, Andean tiger cats may escape from the ocelot effect given the rarity of this mesopredator at higher elevations [5, 25]. This spatial segregation has been found on a horizontal rather than a vertical scale across the Amazon basin for northern tiger cats. In the Guianan savanna shield, northern tiger cats were segregated and used more open and drier habitats even when ocelots occurred at low numbers (0.029 ind./km^2^) and were restricted to some gallery forests and forest patches within savannas [100].

### Conservation implications

The results of this study may help to develop conservation strategies for Andean tiger cat populations thriving in the vanishing cloud forests from the Middle Cauca because our study design represented nearly 67% of their presumed elevational range (1,500–4,800 m asl) in Colombia [19]. A common paradigm when focusing felid research and conservation efforts is to prioritize large species over the needs and threats of smaller ones because of their charisma and cultural value to society [33, 102]. For a long time, the highland forms of the *tigrinus* species complex were not the global, national, and local focus of conservationists because they are small, elusive cats living in harsh conditions, making them difficult to study [103]. Mountain forests worldwide share a common pattern of greater landscape degradation in the lowlands [33]. Most of the degraded lands in the Northern Andes of Colombia are in the lowland dry forests of the Cauca and Magdalena basins, but this trend has been changing since the last century [104]. The increased landscape degradation upward from the mountains together with climate change could lead to important contractions of the Andean tiger cat’s remaining habitat. We need to examine whether Andean tiger cats may experience vertical migrations across their geographic range in the future to resist the rise in temperature and habitat fragmentation. We highlight the potential of this highland felid to be a sentinel species on the impacts of climate and land-use change on the Andean cloud forest, as has been previously proposed for other small carnivores [105].

Supporting species persistence in forest-dwelling predators necessarily involves granting suitable forest conditions on the long run [106]. Endangered Pacific stone martens (*Martes caurina*), for instance, specialize in seeking logs and hollows at live trees in forests to establish resting and denning sites, with a particular tendency to use structures of trees with larger diameters [106]. Our results demonstrate that leading the leaf litter cover to gradually accumulate may improve the quality of the habitat available for the Andean tiger cat, which could be considered in future forest management actions interested in benefiting this threatened felid. Indeed, deep leaf litters are regarded as a key trait of cloud forests worldwide [42], providing important resources for a diversity of taxa [55, 96].

The highest habitat use for Andean tiger cats was obtained in the MPNR, a nature reserve established in 2007 by a local NGO after the end of the armed conflict in the Northwestern Andes [107]. The MPNR protects a key corridor for the Tatamá-Orquídeas national park system and is considered a biodiversity refuge for multiple biological groups [107]. This protected area is currently expanding to new land, and there is a cloud forest restoration program that would be key for the long-term conservation of Andean tiger cats. Nearly 10 km south of the MPNR, the CSJIMD has not suffered the same fate as the MPNR because its administrative policies allow the exploitation of forest resources within the reserve. The lack of government restrictions to prevent the transformation of the Andean cloud forest to land for cultivating avocado crops and cattle pastures in the highlands of the CSJIMD may lead to the disappearance of this important biological corridor. CSJIMD was the site with the second highest habitat use estimates for Andean tiger cats; therefore, it is necessary to prioritize the implementation of more restrictive policies to curb the deforestation that this protected area is currently suffering. To guarantee the long-term conservation of Andean tiger cat populations in this part of the Middle Cauca, it will also be necessary to determine whether there is enough connectivity between the CSJIMD and the MPNR. In the future, we hope that the general public will likely recognize the potential of the Andean tiger cat as a flagship species of the Andean cloud forest.

## Concluding remarks

Our results showed that in our study areas, Andean tiger cat habitat use was mainly modulated by microhabitat structure, elevation, and codetectability with prey and tayras. Additionally, there was a greater probability of finding Andean tiger cats far from human settlements and of detecting them using mainly cameras with the shortest PIR time delays. There was no evidence of competitive exclusion on the habitat use of Andean tiger cats, likely due to the low abundance of intraguild killers/predators in this system, as predicted by the ocelot effect theory. Indeed, the species was more detectable when its intraguild predators were present. The protected areas currently studied may be considered effective barriers against the effects of fragmentation and forest loss on the habitat use of Andean tiger cats in the region. Decision makers should halt the progress of the deforestation frontiers both inside and around protected areas while preserving the vertical structure of the forest ground for the conservation of this mountain felid.

## Supporting information

S1 Fig The results of the principal component analysis performed to summarize four landscape metrics in a 500 m buffer to model the effects of landscape structure on the habitat use of the Andean tiger cat. The proportion of variance explained by each component (A), the weight of each covariate on each component (B), and the coordinate space of the first and second components (C) with a comparison between protected areas (D). The three protected areas in which we obtained the landscape metrics were the Mesenia-Paramillo Nature Reserve (MPNR), the Campoalegre Soil Conservation District (CSCD) and the Cuchilla del San Juan Integrated Management District (CSJIMD). The metrics were: Forest_cov; cloud forest amount, Forest_PCI; patch cohesion index, SHANNON; landscape heterogeneity and EDGE; forest edge density. Dim1 (first component) represents more heterogeneous sites with greater forest edge (positive values) to more forested sites with greater cohesion among patches (negative values).
(DOCX)

S2 Fig Pairwise Pearson’s correlation coefficients for the original set of covariates selected to explain the habitat use of the Andean tiger cat in three protected cloud forests of northwestern Colombia. The final set of numerical covariates used in the occupancy modeling of the Andean tiger cat included LS; landscape structure, AD; canopy height, CD; canopy cover, CH; leaf litter cover, PH; leaf litter depth, slope, DIST_POP; distance to human settlements and elev; elevation. For the modeling of detection probability, the final set of numerical covariates included CF; herbaceous cover, slope, and DAYS; survey effort of the camera traps.
(DOCX)

S3 Fig Posterior predictive checks obtained for the occupancy models incorporating the effect of leaf litter depth (A), quadratic elevation (B), and distance to human settlements (C). The Bayesian P value was calculated by simulating 500 datasets under the MacKenzie-Bailey Goodness of Fit approach and comparing the proportion of simulated zeros against the proportion of observed zeros in the dataset for each model. Matching proportions (red line in the middle of the histogram) and Bayesian P values > 0.05 indicate good model fit.
(DOCX)

S4 Fig Moran’s I spatial correlograms for the residuals in the occupancy component of the three top-ranked models used to predict the habitat use of Andean tiger cats in cloud forests of the Middle Cauca, Colombia. (DOCX)

S1 Table List of covariates and their effect on the habitat use and detection of the Andean tiger cat in cloud forests of the Middle Cauca, Colombia. Ψ; effect on habitat use, p; effect on detection, +; positive effect, -; negative effect, +/-; effect dependent on the type of factor, GIS; geographic information system.
(DOCX)

S2 Table Estimates of the posterior distribution of the occupancy and detection parameters for the Andean tiger cat, including the protected areas as random effects. Considering each protected area individually, the null model for CSCD yielded a 6% chance of detecting the Andean tiger cat if present (p = 0.06, SD = 0.02, CI = 0.02–0.11) and an average occupancy of 63% (Ψ = 0.63, SD = 0.16, CI = 0.32–0.95). In the CSJIMD, the null model showed an average detection of 15% (p = 0.15, SD = 0.02, CI = 0.11–0.19) and an average occupancy of 71% (Ψ = 0.71, SD = 0.11, CI = 0.47–0.92). The null model for MPNR showed an average detection of 18% (p = 0.18, SD = 0.01, CI = 0.14–0.22) and an average occupancy of 85% (Ψ = 0.85, SD = 0.08, CI = 0.65–0.96). Abbreviations: ELPD; expected predictive accuracy, K; number of parameters, ELPD_Δ_; difference in expected predictive accuracy between any model and the best model, SE; standard error of the difference in predictive accuracy.
(DOCX)

S3 Table Estimates of the conditional cooccurrence models evaluating the effects of prey, tayras, and intraguild killers/predators on the habitat use of the Andean tiger cat in three protected cloud forests from the Middle Cauca, Colombia. Abbreviations: AICc; Akaike Information Criterion Value, df; degrees of freedom, AIC_Δ_; difference of the AIC of each model with the best model, and AIC_w_; relative support value of each model.
(DOCX)

S4 Table Locations, survey length, sampling effort (number of days of activity), type of region, sampling area and estimations of occupancy and detection probabilities for other studies carried out on the *tigrinus* species complex throughout South America. (DOCX)
