# Supplementary material for: Assessing microhabitat, landscape features and intraguild relationships in the occupancy of the enigmatic and threatened Andean tiger cat (Leopardus tigrinus pardinoides) in the cloud forests of northwestern Colombia
Source: PLoS One. 2023 Jul 10;18(7):e0288247. doi: 10.1371/journal.pone.0288247 (PMC10332582; doi:10.1371/journal.pone.0288247)
Supplement: S1 Fig — The proportion of variance explained by each component (A), the weight of each covariate on each component (B), and the coordinate space of the first and second components (C) with a comparison between protected areas (D). The three protected areas in which we obtained the landscape metrics were the Mesenia-Paramillo Nature Reserve (MPNR), the Campoalegre Soil Conservation District (CSCD) and the Cuchilla del San Juan Integrated Management District (CSJIMD). The metrics were: Forest_cov; cloud forest amount, Forest_PCI; patch cohesion index, SHANNON; landscape heterogeneity and EDGE; forest edge density. Dim1 (first component) represents more heterogeneous sites with greater forest edge (positive values) to more forested sites with greater cohesion among patches (negative values). (DOCX) [file pone.0288247.s001.docx]

**Assessing microhabitat, landscape features and intraguild relationships in the occupancy of the enigmatic and threatened Andean tiger cat (*Leopardus tigrinus pardinoides*) in the cloud forests of northwestern Colombia**

Juan Camilo Cepeda-Duque, Andrés Montes-Rojas, Gabriel P. Andrade-Ponce, Uriel Rendón-Jaramillo, Valentina López-Velasco, V, Eduven Arango-Correa, Álex M. López-Barrera, Luis Mazariegos, Diego J. Lizcano, Andrés Link & Tadeu G. de Oliveira.

SUPPORTING INFORMATION

S1 Fig.


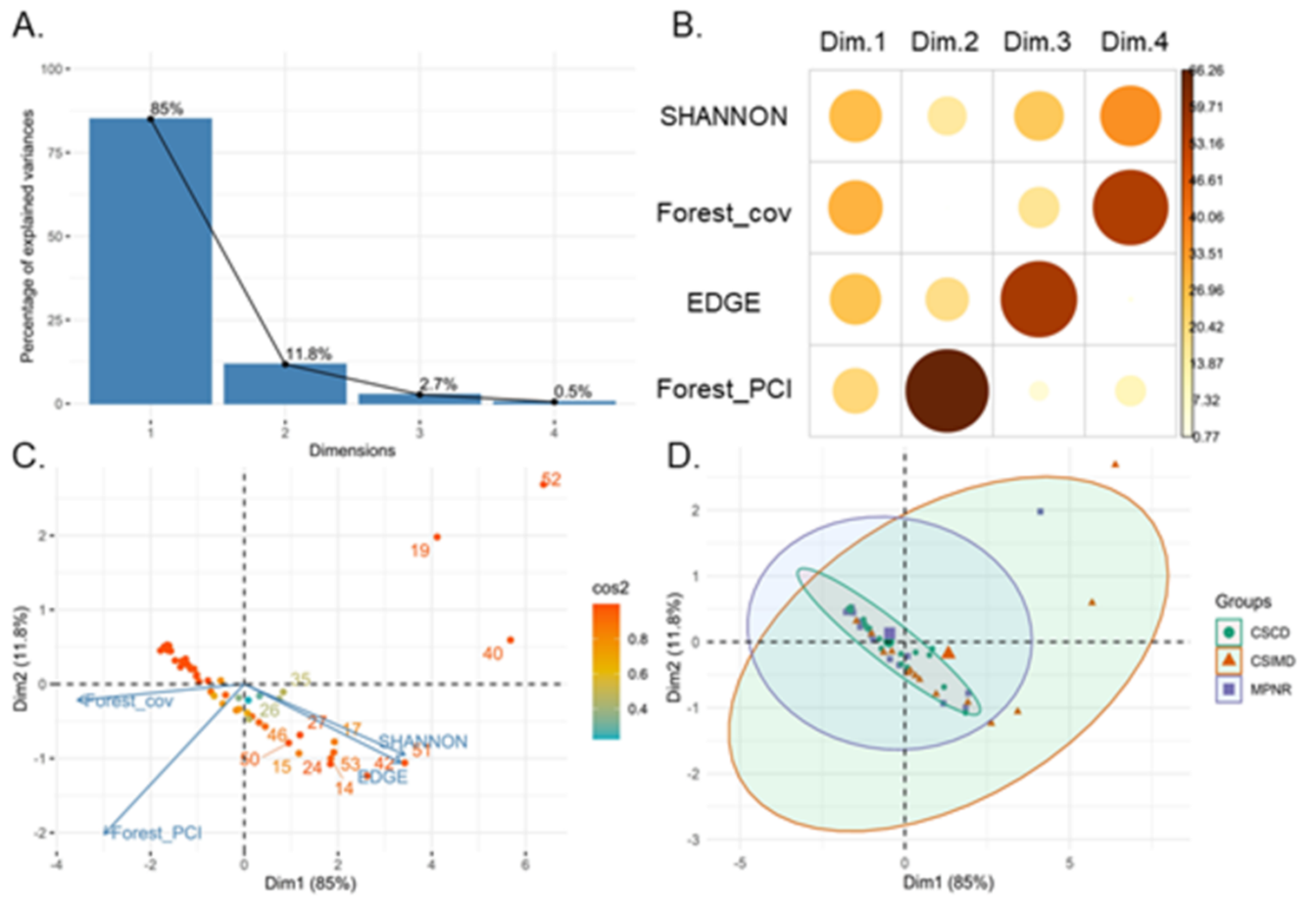


**S1 Fig**. **The results of the principal component analysis performed to summarize four landscape metrics in a 500 m buffer to model the effects of landscape structure on the habitat use of the Andean tiger cat. The proportion of variance explained by each component (A), the weight of each covariate on each component (B), and the coordinate space of the first and second components (C) with a comparison between protected areas (D). The three protected areas in which we obtained the landscape metrics were the Mesenia-Paramillo Nature Reserve (MPNR), the Campoalegre Soil Conservation District (CSCD) and the Cuchilla del San Juan Integrated Management District (CSJIMD).** The metrics were: Forest_cov: cloud forest amount, Forest_PCI: patch cohesion index, SHANNON: landscape heterogeneity and EDGE: forest edge density. Dim1 (first component) represents more heterogeneous sites with greater forest edge (positive values) to more forested sites with greater cohesion among patches (negative values).
