# Supplementary material for: Assessing microhabitat, landscape features and intraguild relationships in the occupancy of the enigmatic and threatened Andean tiger cat (Leopardus tigrinus pardinoides) in the cloud forests of northwestern Colombia
Source: PLoS One. 2023 Jul 10;18(7):e0288247. doi: 10.1371/journal.pone.0288247 (PMC10332582; doi:10.1371/journal.pone.0288247)
Supplement: S2 Fig — The final set of numerical covariates used in the occupancy modeling of the Andean tiger cat included LS; landscape structure, AD; canopy height, CD; canopy cover, CH; leaf litter cover, PH; leaf litter depth, slope, DIST_POP; distance to human settlements and elev; elevation. For the modeling of detection probability, the final set of numerical covariates included CF; herbaceous cover, slope, and DAYS; survey effort of the camera traps. (DOCX) [file pone.0288247.s002.docx]

**Assessing microhabitat, landscape features and intraguild relationships in the occupancy of the enigmatic and threatened Andean tiger cat (*Leopardus tigrinus pardinoides*) in the cloud forests of northwestern Colombia**

Juan Camilo Cepeda-Duque, Andrés Montes-Rojas, Gabriel P. Andrade-Ponce, Uriel Rendón-Jaramillo, Valentina López-Velasco, V, Eduven Arango-Correa, Álex M. López-Barrera, Luis Mazariegos, Diego J. Lizcano, Andrés Link & Tadeu G. de Oliveira.

SUPPORTING INFORMATION

S2 Fig.


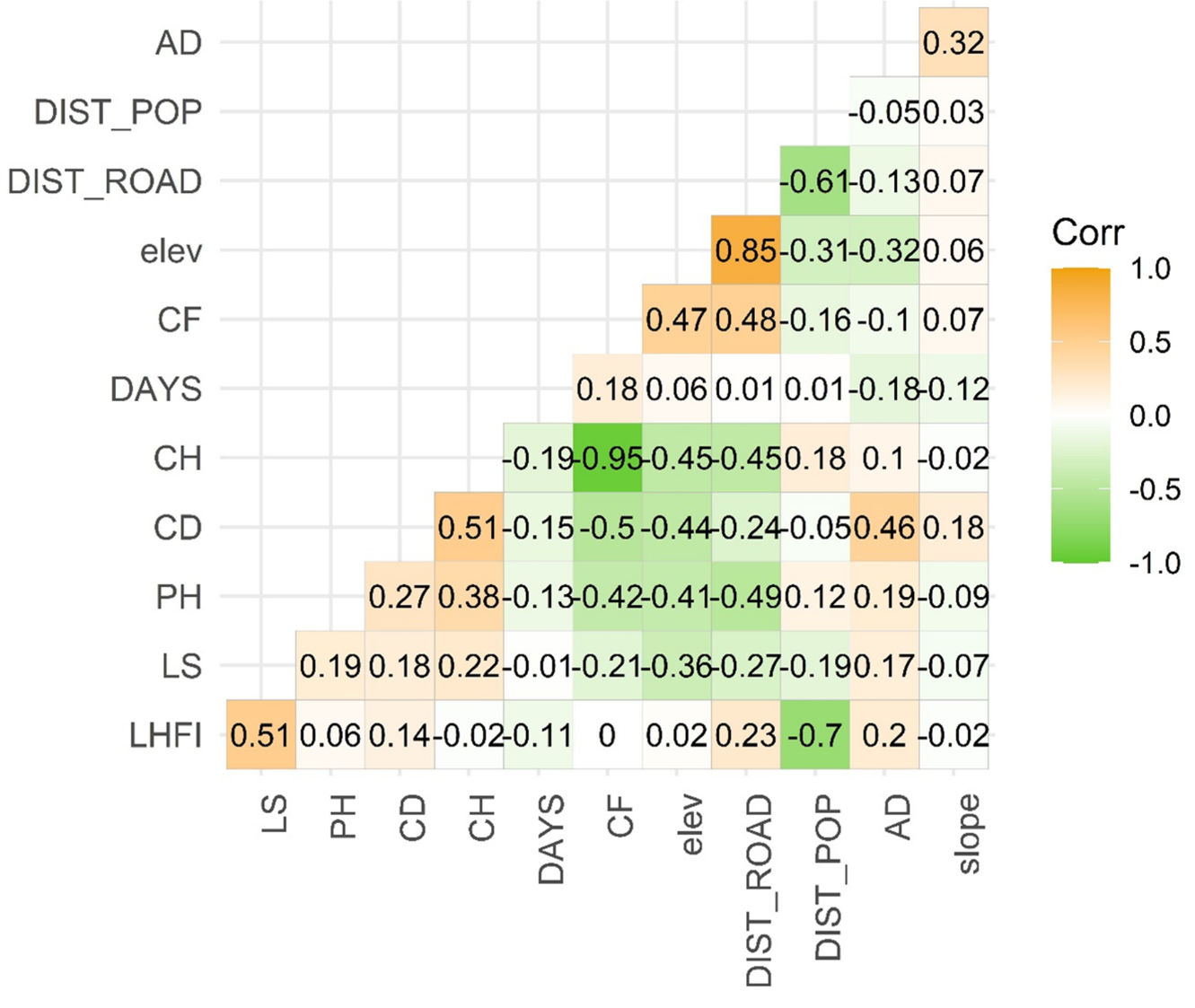


**S2 Fig.** **Pairwise Pearson's correlation coefficients results for the original set of covariates selected to explain the habitat use of the Andean tiger cat in three protected cloud forests of northwestern Colombia**. The final set of numerical covariates for the occupancy modeling of the Andean tiger cat included LS: landscape structure, AD: canopy height, CD: canopy cover, CH: leaf litter cover, PH: leaf litter depth, slope, DIST_POP: distance to human settlements and elev: elevation. For the modeling of detection probability, the final set of numerical covariates included CF: herbaceous cover, slope, and DAYS: survey effort of the camera traps.
