# Supplementary material for: Assessing microhabitat, landscape features and intraguild relationships in the occupancy of the enigmatic and threatened Andean tiger cat (Leopardus tigrinus pardinoides) in the cloud forests of northwestern Colombia
Source: PLoS One. 2023 Jul 10;18(7):e0288247. doi: 10.1371/journal.pone.0288247 (PMC10332582; doi:10.1371/journal.pone.0288247)
Supplement: S3 Fig — Posterior predictive checks obtained for the occupancy models incorporating the effect of leaf litter depth (A), quadratic elevation (B), and distance to human settlements (C). The Bayesian P value was calculated by simulating 500 datasets under the MacKenzie-Bailey Goodness of Fit approach and comparing the proportion of simulated zeros against the proportion of observed zeros in the dataset for each model. Matching proportions (red line in the middle of the histogram) and Bayesian P values > 0.05 indicate good model fit. (DOCX) [file pone.0288247.s003.docx]

**Assessing microhabitat, landscape features and intraguild relationships in the occupancy of the enigmatic and threatened Andean tiger cat (*Leopardus tigrinus pardinoides*) in the cloud forests of northwestern Colombia**

Juan Camilo Cepeda-Duque, Andrés Montes-Rojas, Gabriel P. Andrade-Ponce, Uriel Rendón-Jaramillo, Valentina López-Velasco, V, Eduven Arango-Correa, Álex M. López-Barrera, Luis Mazariegos, Diego J. Lizcano, Andrés Link & Tadeu G. de Oliveira.

**SUPPORTING INFORMATION**

S3 Fig.


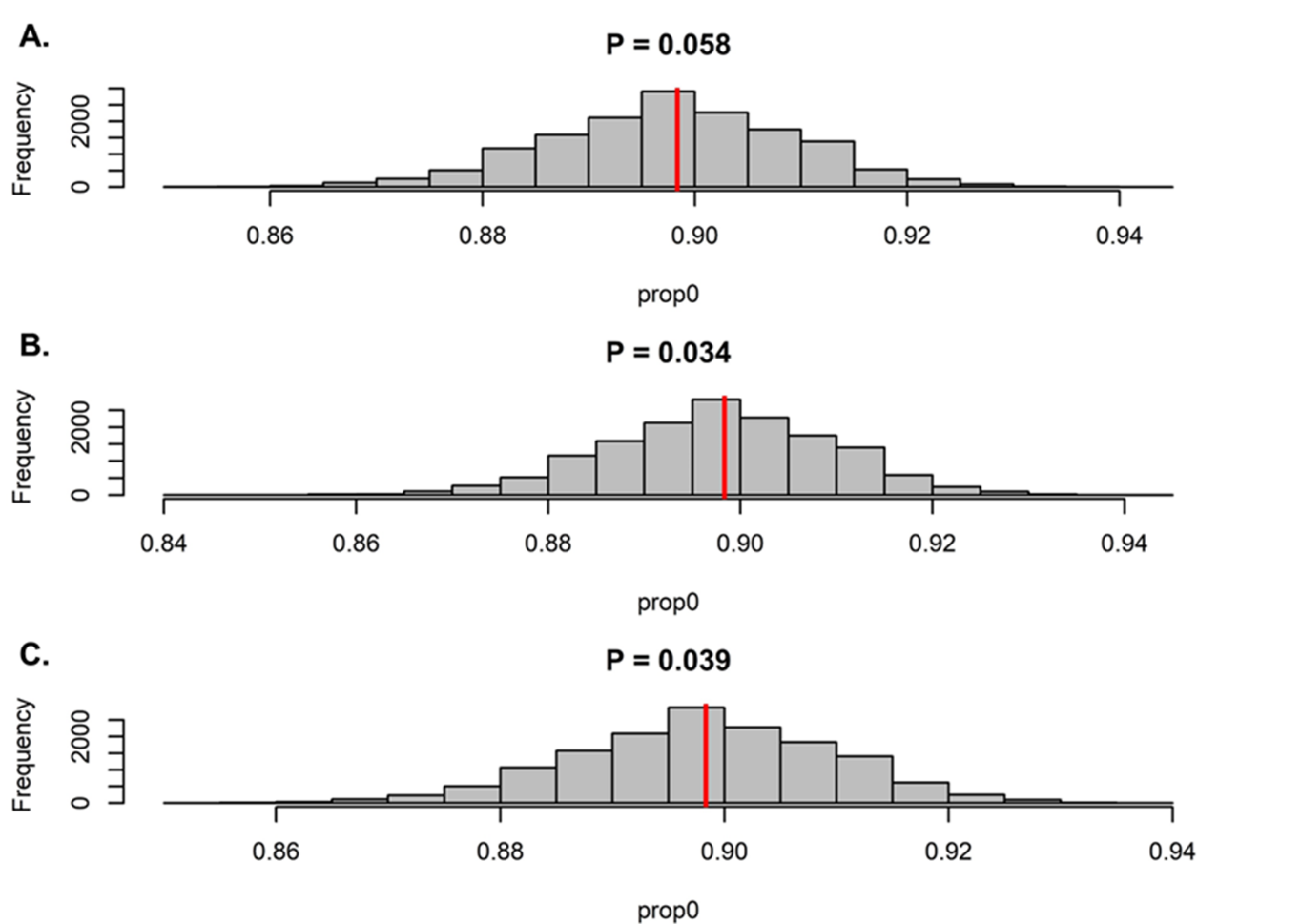


**S3 Fig**. **Posterior predictive checks obtained for the occupancy models incorporating the effect of leaf litter depth (A), quadratic elevation (B), and distance to human settlements (C). The Bayesian P value was calculated by simulating 500 datasets under the MacKenzie-Bailey Goodness of Fit approach and comparing the proportion of simulated zeros against the proportion of observed zeros in the dataset for each model.** The matching proportions (red line in the middle of the histogram) and Bayesian P values > 0.05 indicate good model fit.
