# Supplementary material for: Assessing microhabitat, landscape features and intraguild relationships in the occupancy of the enigmatic and threatened Andean tiger cat (Leopardus tigrinus pardinoides) in the cloud forests of northwestern Colombia
Source: PLoS One. 2023 Jul 10;18(7):e0288247. doi: 10.1371/journal.pone.0288247 (PMC10332582; doi:10.1371/journal.pone.0288247)
Supplement: S4 Fig — (DOCX) [file pone.0288247.s004.docx]

**Assessing microhabitat, landscape features and intraguild relationships in the occupancy of the enigmatic and threatened Andean tiger cat (*Leopardus tigrinus pardinoides*) in the cloud forests of northwestern Colombia**

Juan Camilo Cepeda-Duque, Andrés Montes-Rojas, Gabriel P. Andrade-Ponce, Uriel Rendón-Jaramillo, Valentina López-Velasco, V, Eduven Arango-Correa, Álex M. López-Barrera, Luis Mazariegos, Diego J. Lizcano, Andrés Link & Tadeu G. de Oliveira.

**SUPPORTING INFORMATION**

S4 Fig.


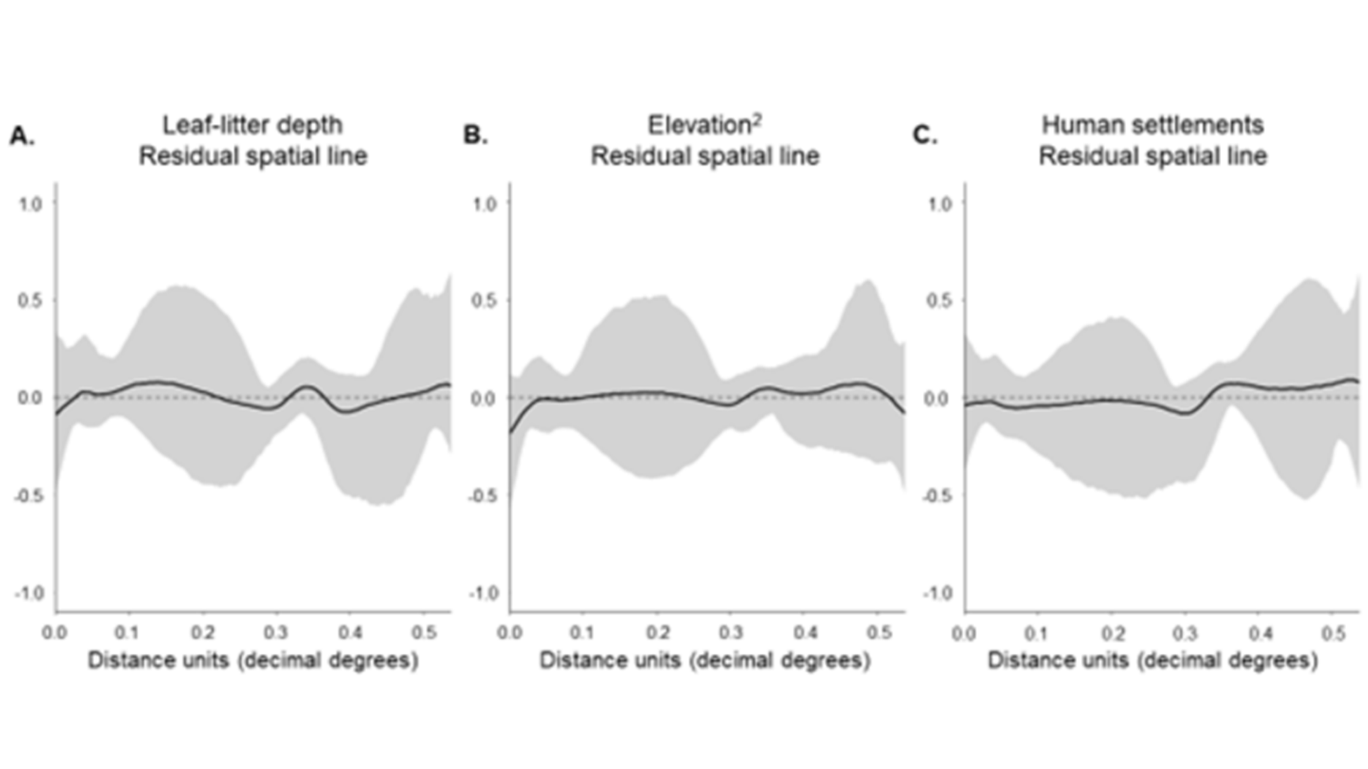


**S4 Fig. Moran's I spatial correlograms for the residuals in the occupancy component of the three top-ranked models used to predict the habitat use of Andean tiger cats in cloud forests of the Middle Cauca, Colombia.**
