# Supplementary material for: Assessing microhabitat, landscape features and intraguild relationships in the occupancy of the enigmatic and threatened Andean tiger cat (Leopardus tigrinus pardinoides) in the cloud forests of northwestern Colombia
Source: PLoS One. 2023 Jul 10;18(7):e0288247. doi: 10.1371/journal.pone.0288247 (PMC10332582; doi:10.1371/journal.pone.0288247)
Supplement: S1 Table — Ψ; effect on habitat use, p; effect on detection, +; positive effect, -; negative effect, +/-; effect dependent on the type of factor, GIS; geographic information system. (DOCX) [file pone.0288247.s005.docx]

**Assessing microhabitat, landscape features and intraguild relationships in the occupancy of the enigmatic and threatened Andean tiger cat (*Leopardus tigrinus pardinoides*) in the cloud forests of northwestern Colombia**

Juan Camilo Cepeda-Duque, Gabriel P. Andrade-Ponce, Andrés Montes-Rojas, Uriel Rendón-Jaramillo, Valentina López-Velasco, V, Eduven Arango-Correa, Álex M. López-Barrera, Luis Mazariegos, Diego J. Lizcano, Andrés Link & Tadeu G. de Oliveira.

**SUPPORTING INFORMATION**

**S1 TABLE.**

**S1 Table**. **List of covariates and their effect on the habitat use and detection of the Andean tiger cat in cloud forests of the Middle Cauca, Colombia.** Ψ = effect on habitat use; p = effect on detection; + = positive effect; - = negative effect; +/- = effect dependent on the type of factor; GIS = geographic information system.

| **Scale** | **Covariate (abbreviation)** | **Measure unit** | **Parameter** | **Effect** | **Source** |
| --- | --- | --- | --- | --- | --- |
| Site | Canopy cover (CD) | % | Ψ | + | Freitas et al., (2002). |
|  | Leaf-litter depth (PH) | Cm | Ψ | + | Field |
|  | Canopy height (AD) | M | Ψ | + |  |
|  | Leaf-litter cover (CH)^a^ | % | Ψ | + |  |
|  | Slope (SL) | Degrees | Ψ/p | - |  |
|  | Herbaceous cover (CF) | % | p | - |  |
|  | Camera trap effort (E) | Number of days | P | + |  |
|  | PIR time delay (PIR) | Factor | P | - |  |
| Landscape | Distance to roads (ROADS)^b^ | km | Ψ | - | GIS |
|  | Distance to human settlements (HUM) | km | Ψ | - | GIS |
|  | Legacy Human Footprint Index (LFHI)^b^ | % | Ψ | + | Ayram et al., (2020) |
|  | Forest cover | Ha | Ψ | - | González-González et al., (2022) |
|  | Edge density | Ha | Ψ | - |  |
|  | Patch Cohesion Index | Ha | Ψ | + |  |
|  | Landscape heterogeneity – Shannon Index | Bits | Ψ | - |  |
|  | Elevation | M | Ψ | + | Field |
|  | Landscape structure^c^ (LS) | Eigenvalues of the first Principal Component | Ψ | - | Analysis |
|  | Protected area (PA) | Factor | Ψ | +/- | Field |

^a^ Highly correlated covariates (r ≥ 0.65) removed from the detection modeling.
^b^ Highly correlated covariates (r ≥ 0.65) removed from the habitat use modeling.
^c^ Principal component analysis performed for the landscape structure covariates.
