# Supplementary material for: Assessing microhabitat, landscape features and intraguild relationships in the occupancy of the enigmatic and threatened Andean tiger cat (Leopardus tigrinus pardinoides) in the cloud forests of northwestern Colombia
Source: PLoS One. 2023 Jul 10;18(7):e0288247. doi: 10.1371/journal.pone.0288247 (PMC10332582; doi:10.1371/journal.pone.0288247)
Supplement: S2 Table — Considering each protected area individually, the null model for CSCD yielded a 6% chance of detecting the Andean tiger cat if present (p = 0.06, SD = 0.02, CI = 0.02–0.11) and an average occupancy of 63% (Ψ = 0.63, SD = 0.16, CI = 0.32–0.95). In the CSJIMD, the null model showed an average detection of 15% (p = 0.15, SD = 0.02, CI = 0.11–0.19) and an average occupancy of 71% (Ψ = 0.71, SD = 0.11, CI = 0.47–0.92). The null model for MPNR showed an average detection of 18% (p = 0.18, SD = 0.01, CI = 0.14–0.22) and an average occupancy of 85% (Ψ = 0.85, SD = 0.08, CI = 0.65–0.96). Abbreviations: ELPD; expected predictive accuracy, K; number of parameters, ELPDΔ; difference in expected predictive accuracy between any model and the best model, SE; standard error of the difference in predictive accuracy. (DOCX) [file pone.0288247.s006.docx]

**Assessing microhabitat, landscape features and intraguild relationships in the occupancy of the enigmatic and threatened Andean tiger cat (*Leopardus tigrinus pardinoides*) in the cloud forests of northwestern Colombia**

Juan Camilo Cepeda-Duque, Andrés Montes-Rojas, Gabriel P. Andrade-Ponce, Uriel Rendón-Jaramillo, Valentina López-Velasco, V, Eduven Arango-Correa, Álex M. López-Barrera, Luis Mazariegos, Diego J. Lizcano, Andrés Link & Tadeu G. de Oliveira.

**SUPPORTING INFORMATION**

**S2 TABLE.**

**S2 Table. Estimates of the posterior distribution of the occupancy and detection parameters for the Andean tiger cat, including the protected areas as random effects.** **Considering each protected area individually, the null model for CSCD yielded a 6% chance to obtain a detection of the Andean tiger cat if present (p = 0.06, SD = 0.02, CI = 0.02 – 0.11) and an average occupancy of 63 % (Ψ = 0.63, SD = 0.16, CI = 0.32 – 0.95). In the CSJIMD, the null model showed an average detection of 15 % (p = 0.15, SD = 0.02, CI = 0.11 – 0.19) and an average occupancy of 71 % (Ψ = 0.71, SD = 0.11, CI = 0.47 – 0.92). The null model for MPNR showed an average detection of 18 % (p = 0.18, SD = 0.01, CI = 0.14 – 0.22) and an average occupancy of 85 % (Ψ = 0.85, SD = 0.08, CI = 0.65 – 0.96**). Abbreviations: ELPD; expected predictive accuracy, K; number of parameters, ELPD_Δ_; difference in expected predictive accuracy between any model and the best model, SE; standard error of the difference in predictive accuracy, CSCD; Campoalegre Soil Conservation District, CSJIMD; Cuchilla del San Juan Integrated Management District, MPNR; Mesenia-Paramillo Nature Reserve.

| Model | ELPD | K | ELPD_Δ_ | SE ELPD | ELPD weight |
| --- | --- | --- | --- | --- | --- |
| p(PIR)Ψ(PH) | -388.967 | 11.825 | 0 | 0 | 0.291 |
| p(PIR)Ψ(PH\|PA) | -389.006 | 12.876 | -0.038 | 0.884 | 0.002 |
| p(PIR)Ψ(Elevation^2) | -389.842 | 13.068 | -0.875 | 2.735 | 0.216 |
| p(PIR)Ψ(Elevation^2\|PA) | -389.98 | 13.901 | -1.013 | 2.754 | 0.01 |
| p(PIR)Ψ(HUM) | -391.347 | 11.835 | -2.38 | 3.43 | 0.227 |
| p(PIR)Ψ(CD\|PA) | -391.748 | 13.583 | -2.781 | 3.401 | 0.135 |
| p(PIR)Ψ(HUM\|PA) | -391.78 | 13.01 | -2.813 | 3.298 | 0 |
| p(PIR)Ψ(SL\|PA) | -391.991 | 14.059 | -3.024 | 3.455 | 0.002 |
| p(PIR)Ψ(CH\|PA) | -392.023 | 13.403 | -3.056 | 2.626 | 0.02 |
| p(PIR)Ψ(AD\|PA) | -392.481 | 13.404 | -3.514 | 2.849 | 0 |
| p(PIR)Ψ(CH) | -392.521 | 12.539 | -3.554 | 2.535 | 0 |
| p(PIR)Ψ(Elevation\|PA) | -392.921 | 14.063 | -3.954 | 3.254 | 0 |
| p(PIR)Ψ(LS\|PA) | -393.057 | 14.151 | -4.09 | 2.697 | 0 |
| p(PIR)Ψ(Elevation) | -393.143 | 13.076 | -4.176 | 3.175 | 0 |
| p(PIR)Ψ(CD) | -393.523 | 12.952 | -4.556 | 2.969 | 0 |
| p(PIR)Ψ(AD) | -393.581 | 11.891 | -4.614 | 2.566 | 0 |
| p(PIR)Ψ(SL) | -393.906 | 12.567 | -4.939 | 2.918 | 0 |
| p(PIR)Ψ(LS) | -393.959 | 12.778 | -4.992 | 2.424 | 0 |
| p(.)Ψ(.) | -396.19 | 3.731 | -7.223 | 6.589 | 0.097 |
