# Supplementary material for: Assessing microhabitat, landscape features and intraguild relationships in the occupancy of the enigmatic and threatened Andean tiger cat (Leopardus tigrinus pardinoides) in the cloud forests of northwestern Colombia
Source: PLoS One. 2023 Jul 10;18(7):e0288247. doi: 10.1371/journal.pone.0288247 (PMC10332582; doi:10.1371/journal.pone.0288247)
Supplement: S3 Table — Abbreviations: AICc; Akaike Information Criterion Value, df; degrees of freedom, AICΔ; difference of the AIC of each model with the best model, and AICw; relative support value of each model. (DOCX) [file pone.0288247.s007.docx]

**Assessing microhabitat, landscape features and intraguild relationships in the occupancy of the enigmatic and threatened Andean tiger cat (*Leopardus tigrinus pardinoides*) in the cloud forests of northwestern Colombia**

Juan Camilo Cepeda-Duque, Andrés Montes-Rojas, Gabriel P. Andrade-Ponce, Uriel Rendón-Jaramillo, Valentina López-Velasco, V, Eduven Arango-Correa, Álex M. López-Barrera, Luis Mazariegos, Diego J. Lizcano, Andrés Link & Tadeu G. de Oliveira.

**SUPPORTING INFORMATION**

**S3 TABLE.**

**S3 Table**. **Estimates of the conditional co-occurrence models evaluating the effects of prey, tayras, and potential intraguild killers on the habitat use of the Andean tiger cat in three protected cloud forests from the Middle Cauca, Colombia**. Abbreviations: AICc; Akaike Information Criterion Value, df; degrees of freedom, AIC_Δ_; difference of the AIC of each model with the best model; and AIC_w_; relative support value of each model.

| Model | df | AICc | AIC_Δ_ | AIC_w_ |
| --- | --- | --- | --- | --- |
| Prey |  |  |  |  |
| ΨA,ΨBA=ΨBa,pA,rA,pB,rBa,Rba | 7 | 2157.865 | 0 | 0.15 |
| ΨA,ΨBA=ΨBa,pA,rA,pB,rB | 6 | 2158.176 | 0.312 | 0.128 |
| ΨA,ΨBA,ΨBa, pA,rA,pB,rBa,rBA | 8 | 2158.84 | 0.976 | 0.092 |
| ΨA,ΨBA(PH),ΨBa, pA,rA,pB,rBa,rBA | 8 | 2158.84 | 0.976 | 0.092 |
| ΨA,ΨBA(HUM),ΨBa, pA,rA,pB,rBa,rBA | 8 | 2158.84 | 0.976 | 0.092 |
| ΨA,ΨBA(Elevation^2^),ΨBa, pA,rA,pB,rBa,rBA | 8 | 2158.84 | 0.976 | 0.092 |
| ΨA,ΨBA,ΨBa,pA,rA,pB,rB | 7 | 2158.92 | 1.055 | 0.088 |
| ΨA,ΨBA,ΨBa (PH),pA,rA,pB,rB | 7 | 2158.92 | 1.055 | 0.088 |
| ΨA,ΨBA,ΨBa (HUM),pA,rA,pB,rB | 7 | 2158.92 | 1.055 | 0.088 |
| ΨA,ΨBA,ΨBa (Elevation^2^),pA,rA,pB,rB | 7 | 2158.92 | 1.055 | 0.088 |
| ΨA,ΨBA,ΨBa,pA=rA,pB=rB | 5 | 2216.597 | 58.732 | 0 |
| ΨA,ΨBA(PH),ΨBa,pA=rA,pB=rB | 5 | 2216.597 | 58.732 | 0 |
| ΨA,ΨBA(HUM),ΨBa,pA=rA,pB=rB | 5 | 2216.597 | 58.732 | 0 |
| ΨA,ΨBA(Elevation^2^),ΨBa,pA=rA,pB=rB | 5 | 2216.597 | 58.732 | 0 |
| ΨA,ΨBA=ΨBa,pA=rA,pB=rB | 4 | 2218.353 | 60.489 | 0 |
| Tayras |  |  |  |  |
| ΨA,ΨBA=ΨBa,pA,rA,pB,rBa,rBA | 7 | 1630.013 | 0 | 0.475 |
| ΨA,ΨBA,ΨBa,pA,rA,pB,rBa,rBA | 8 | 1632.582 | 2.569 | 0.131 |
| ΨA,ΨBA,ΨBa(PH),pA,rA,pB,rBa,rBA | 8 | 1632.582 | 2.569 | 0.131 |
| ΨA,ΨBA,ΨBa(HUM),pA,rA,pB,rBa,rBA | 8 | 1632.582 | 2.569 | 0.131 |
| ΨA,ΨBA,ΨBa(Elevation^2^),pA,rA,pB,rBa,rBA | 8 | 1632.582 | 2.569 | 0.131 |
| ΨA,ΨBA=ΨBa,pA,rA,pB,rB | 6 | 1646.77 | 16.757 | 0 |
| ΨA,ΨBA,ΨBa,pA,rA,pB,rB | 7 | 1649.263 | 19.25 | 0 |
| ΨA,ΨBA,ΨBa,pA=rA,pB=rB | 5 | 1651.017 | 21.004 | 0 |
| ΨA,ΨBA(PH),ΨBa,pA=rA,pB=rB | 5 | 1651.017 | 21.004 | 0 |
| ΨA,ΨBA(HUM),ΨBa,pA=rA,pB=rB | 5 | 1651.017 | 21.004 | 0 |
| ΨA,ΨBA(Elevation^2^),ΨBa,pA=rA,pB=rB | 5 | 1651.017 | 21.004 | 0 |
| ΨA,ΨBA=ΨBa,pA=rA,pB=rB | 4 | 1651.409 | 21.395 | 0 |
| Intraguild killers |  |  |  |  |
| ΨA,ΨBA=ΨBa,pA,rA,pB,rB | 6 | 1386.497 | 0 | 1 |
| ΨA,ΨBA=ΨBa,pA,rA,pB,rBa,rBA | 7 | 1388.656 | 2.16 | 0.34 |
| ΨA,ΨBA,ΨBa,pA,rA,pB,rB | 7 | 1388.997 | 2.5 | 0.287 |
| ΨA,ΨBA,ΨBa,pA,rA,pB,rBa,rBA | 8 | 1391.265 | 4.768 | 0.092 |
| ΨA,ΨBA,ΨBa(PH),pA,rA,pB,rBa,rBA | 8 | 1391.265 | 4.768 | 0.092 |
| ΨA,ΨBA,ΨBa(HUM),pA,rA,pB,rBa,rBA | 8 | 1391.265 | 4.768 | 0.092 |
| ΨA,ΨBA,ΨBa(Elevation^2),pA,rA,pB,rBa,rBA | 8 | 1391.265 | 4.768 | 0.092 |
| ΨA,ΨBA=ΨBa,pA=rA,pB=rB | 4 | 1404.688 | 18.191 | 0 |
| ΨA,ΨBA,ΨBa,pA=rA,pB=rB | 5 | 1405.122 | 18.625 | 0 |
| ΨA,ΨBA(PH),ΨBa,pA=rA,pB=rB | 5 | 1405.122 | 18.625 | 0 |
| ΨA,ΨBA(HUM),ΨBa,pA=rA,pB=rB | 5 | 1405.122 | 18.625 | 0 |
| ΨA,ΨBA(Elevation^2^),ΨBa,pA=rA,pB=rB | 5 | 1405.122 | 18.625 | 0 |
