# Supplementary material for: Assessing microhabitat, landscape features and intraguild relationships in the occupancy of the enigmatic and threatened Andean tiger cat (Leopardus tigrinus pardinoides) in the cloud forests of northwestern Colombia
Source: PLoS One. 2023 Jul 10;18(7):e0288247. doi: 10.1371/journal.pone.0288247 (PMC10332582; doi:10.1371/journal.pone.0288247)
Supplement: S4 Table — (DOCX) [file pone.0288247.s008.docx]

**Assessing microhabitat, landscape features and intraguild relationships in the occupancy of the enigmatic and threatened Andean tiger cat (*Leopardus tigrinus pardinoides*) in the cloud forests of northwestern Colombia**

Juan Camilo Cepeda-Duque, Andrés Montes-Rojas, Gabriel P. Andrade-Ponce, Uriel Rendón-Jaramillo, Valentina López-Velasco, V, Eduven Arango-Correa, Álex M. López-Barrera, Luis Mazariegos, Diego J. Lizcano, Andrés Link & Tadeu G. de Oliveira.

**SUPPORTING INFORMATION**

**S4 TABLE.**

**S4 Table. Locations, survey length, sampling effort (number of days of activity), type of region, sampling area and estimations of probabilities of occupation and detection of other studies carried out on the tiger cat species complex throughout South America.**

| **Location** | **Survey length** | **Sampling effort** | **Species** | **Area (km^2^)** | **Ѱ** | **p** | **Reference** |
| --- | --- | --- | --- | --- | --- | --- | --- |
| Rio Grande do Norte, Caatinga, Brazil | May to September2014 | 7,263 | *L. t. tigrinus* | 1,052 | 0.46 | 0.21 | [1] |
| Upper Paraná Atlantic Forest, Argentin | May 2013 to December 2014 | 9,171 | *L. guttulus* | 2,683 | 0.53 | 0.07 | [2] |
| Michelin Ecological Reserve, Atlantic Forest, Brazil | February 2013 to January 2014 | 7,954 | *L. guttulus* | 42 | 0.66 | 0.10 | [3] |
| Boqueirão da Onça, Caatinga, Brazil | January to July 2017 | 8,678 | *L. t. tigrinus* | 600 | 0.64 | 0.08 | [4] |
| Mesenia-Paramillo Nature Reserve, Northwestern Andean cloud forest, Colombia | March to July 2016 – September to July 2019 | 3,625 | *L. t. pardinoides* | 35 | 0.51 | 0.19 | [5] |
| Serranía de los Paraguas, Northwestern Andean cloud forest, Colombia | July 2016 to July 2018 | 23,666 | *L. t. pardinoides* | >7 | 0.73 | 0.01 | [6] |
| Tamandua Ranch, Caatinga Brazil. | January 2010 to February 2014 | 15,748 | *L. t. tigrinus* | 30.7 | 0.60 | 0.4 | [7] |
| Tamandua Ranch, Caatinga, Brazil | August 2018 to June 2021 | 8,566 | *L. t. tigrinus* | 19.7 | 0.62 | 0.22 | [8] |
| Mesenia-Paramillo Nature Reserve, Cuchilla del San Juan Integrated Management District, Campoalegre Soil Conservation District, Northwestern Andean cloud forest, Colombia | December 2020 to June 2022 | 10,689 | *L. t. pardinoides* | 74 | 0.72 | 0.13 | This study |

**References**

[1] Marinho PH, Bezerra D, Antongiovanni M, Fonseca CR, Venticinque EM. Estimating occupancy of the Vulnerable northern tiger cat *Leopardus tigrinus* in Caatinga drylands. Mamm Res 2018;63:33–42. https://doi.org/10.1007/s13364-017-0330-4.

[2] Cruz P, Iezzi ME, de Angelo C, Varela D, di Bitetti MS, Paviolo A. Effects of human impacts on habitat use, activity patterns and ecological relationships among medium and small felids of the Atlantic Forest. PLoS One 2018;13. https://doi.org/10.1371/journal.pone.0200806.

[3] Dechner A, Flesher KM, Lindell C, de Oliveira TV, Maurer BA. Determining carnivore habitat use in a rubber/forest landscape in Brazil using multispecies occupancy models. PLoS One 2018;13. https://doi.org/10.1371/journal.pone.0195311.

[4] Dias D de M, Lima Massara R, de Campos CB, Henrique Guimarães Rodrigues F. Human activities influence the occupancy probability of mammalian carnivores in the Brazilian Caatinga. Biotropica 2019;51. https://doi.org/10.1111/btp.12628.

[5] Bonilla-Sánchez A, Gómez-Ruíz DA, Botero-Cañola S, Rendón-Jaramillo U, Ledesma-Castañeda E, Solari S. Riqueza y monitoreo de mamíferos en áreas protegidas privadas en Antioquia, Colombia. Mastozool Neotrop 2020;27. https://doi.org/10.31687/saremmn.20.27.2.0.11.

[6] Bedoya-Durán MJ, Murillo-García OE, Branch LC. Factors outside privately protected areas determine mammal assemblages in a global biodiversity hotspot in the Andes. Glob Ecol Conserv 2021;32. https://doi.org/10.1016/j.gecco.2021.e01921.

[7] Fox-Rosales LA, de Oliveira TG. Habitat use patterns and conservation of small carnivores in a human-dominated landscape of the semiarid Caatinga in Brazil. Mammalian Biology 2022;102:465–75. https://doi.org/10.1007/s42991-022-00245-3.

[8] Fox-Rosales LA, de Oliveira TG. Interspecific patterns of small cats in an intraguild-killer free area of the threatened Caatinga drylands, Brazil. PLoS One 2023;18:e0284850. https://doi.org/10.1371/journal.pone.0284850.
